# Multifunctional Tailoring of Fertilizer Composites Directly Derived From Phosphate Rock

**DOI:** 10.1002/advs.202517533

**Published:** 2025-12-23

**Authors:** Zenglian Qi, Jianchao Wang, Lulu Chen, Zhenya Lu, Guodong Wang, Hang Ma, Cuihong Hou, Xinxin Wang, Wenqi Ma, Changzhou Wei, Jianbo Shen, Fusuo Zhang, Minghao Zhuang, Chengdong Huang

**Affiliations:** ^1^ State Key Laboratory of Nutrient Use and Management Key Laboratory of Plant‐Soil Interactions Ministry of Education National Academy of Agriculture Green Development National Observation and Research Station of Agriculture Green Development (Quzhou, Hebei) College of Resources and Environmental Sciences China Agricultural University Beijing 100193 China; ^2^ Research and Development Center, Yunnan Yuntianhua Co., Ltd Kunming 650228 China; ^3^ School of Chemical Engineering Zhengzhou University Zhengzhou 450001 China; ^4^ College of Horticulture Hebei Agricultural University Baoding 071001 China; ^5^ College of Resources and Environmental Science Hebei Agricultural University Baoding 071001 China; ^6^ Agricultural College Shihezi University Shihezi 832000 China; ^7^ State Key Laboratory of Urban and Regional Ecology Research Center for Eco‐Environmental Sciences Chinese Academy of Sciences Beijing 100085 China

**Keywords:** calcium, fertilizer composites, magnesium, multifunctional tailoring

## Abstract

To address the critical challenges of depleting high‐grade phosphate reserves and underutilization of calcium (Ca) and magnesium (Mg) resources in low‐grade phosphate rock (LPR), herein, a novel phosphorus‐sulfur mixed acid with urea (PSU) activation system for LPR to fabricate multifunctional fertilizer composites (MFCs) is developed. It is found that under optimized conditions, the activation efficiencies for P, Ca, and Mg reached 78.5%–98.3%. The characterization results including particle size analysis, scanning electron microscopy, and nitrogen adsorption‐desorption isotherms indicated that after activation, MFCs possessed porous structures, endowing it with great adsorption capacity for nutrients. In this regard, MFCs exhibited superior slow‐release properties, significantly reducing Ca and Mg leaching to 27.6% and 73.9%, respectively. In contrast to conventional fertilizers, MFCs substantially increased Chinese cabbage biomass by 11.5%–23.4% in pot experiments. Furthermore, the MFCs are shown to be environmentally friendly, posing no risk of heavy metal contamination to either soil or crops. Overall, this novel activation method developed in this study not only facilitated minimizing the discharge of industrial phosphorus by‐products, but also remediating soil acidity and nutrient deficits caused by long‐term NPK fertilization, offering a sustainable paradigm for integrated nutrient management and acidic soil amelioration.

## Graphical abstract

A novel phosphorus‑sulfur mixed acid with urea (PSU) activation system converts low‑grade phosphate rock (LPR) into multifunctional fertilizer composites (MFCs). MFCs feature porous structures, high nutrient‑activation efficiency, superior slow‑release, and enhanced crop growth, offering a sustainable solution for integrated nutrient management and acidic soil amelioration.

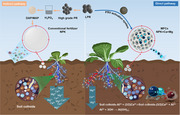

## 1 Introduction

Phosphorus (P), a critical resource for human development primarily derived from phosphate rock (PR), is indispensable for achieving key Sustainable Development Goals (SDGs), including agricultural sustainability, clean energy transition, and environmental pollution control.^[^ ^1^, ^2^, ^3^ ^]^ The demand for P has grown rapidly due to its extensively widespread application in agriculture, chemistry, electronics, new energy, and biomedicine.^[^ ^4^, ^5^, ^6^ ^]^ According to the United States Geological Survey, global PR production has risen significantly from 137 million tons in 1996 to 240 million tons in 2024.^[^ ^7^, ^8^ ^]^ It is predicted that by 2040, global P supply will be insufficient to meet demand.^[^ ^9^ ^]^ However, given that high‐grade PR is becoming increasingly scarce and its grade is persistently declining, particularly in China, the focus of P resource development has shifted to low‐grade phosphate rock (LPR) with a P_2_O_5_ content of 12%–25%.^[^ ^10^ ^]^ On the one hand, China's PR reserves are 3.7 billion tons, accounting for 4.7% of the global total in 2024. On the other hand, over 80% of China's PR reserves are LPR with an average P_2_O_5_ content of 16.9%, substantially lower than the average content of 33% in Moroccan PR.^[^ ^10^, ^11^, ^12^ ^]^ Furthermore, during PR processing, the P utilization efficiency is extremely low, which exacerbates the P resource crisis. Simultaneously, the process generates substantial amounts of by‐products, such as tailings, phosphogypsum, and waste acids.^[^ ^13^ ^]^ These by‐products not only raise increasingly severe environmental concerns, but also result in substantial wastage of critical resources.^[^ ^9^, ^14^ ^]^ For example, significant quantities of P, Ca, and Mg in these by‐products, as essential nutrients, play critical roles in agricultural production.^[^ ^15^, ^16^ ^]^ Therefore, it is imperative to develop highly efficient, green, and sustainable methods for LPR utilization toward zero waste, this is crucial for achieving SDGs related to industry, agriculture, and the environment.

In various PR applications, ≈80%–95% is consumed by agricultural production in the form of fertilizer composites.^[^ ^10^, ^17^ ^]^ Generally, two main pathways have been developed to produce fertilizer composites using PR, including the indirect and direct pathways. The currently dominant method is the indirect pathway, or wet‐process phosphoric acid (H_3_PO_4_) technology. It involves producing H_3_PO_4_ before converting it into fertilizer composites for agricultural use. A major disadvantage of this process is that it consumes a large amount of chemicals and generates substantial phosphate by‐products.^[^ ^9^, ^14^, ^18^ ^]^ To address the shortcomings in the indirect pathway, the direct pathway using PR to directly produce fertilizer composites has emerged as a research hotspot. This requires the direct conversion of the “passivated” to “activated” resources in PR, as prior procedures. Recently, several methods have been proposed, including mechanochemical,^[^ ^19^, ^20^ ^]^ high‐temperature,^[^ ^21^ ^]^ strong acid activation^[^ ^22^ ^]^ and other methods.^[^ ^23^, ^24^ ^]^ Although these methods achieve partial resource activation, they are invariably constrained by fundamental limitations. For example, mechanochemical activation suffers from high energy consumption and incomplete structural decomposition,^[^ ^25^ ^]^ and the high‐temperature methods require extreme energy inputs, typically operating at 1250–1350 °C.^[^ ^26^ ^]^ Strong acid activation using sulfuric acid (H_2_SO_4_) or nitric acid (HNO_3_) exhibits merits such as a high resource activation rate and low energy consumption. However, this approach has notable drawbacks, including high acid consumption, the generation of massive amounts of gypsum, and low overall resource use efficiency.^[^ ^13^ ^]^ Therefore, the efficient and environmentally sustainable utilization of PR remains technically challenging via current direct activation methods. Proposing innovative methods toward full use of PR resources, including P, Ca and Mg wasted in current methods, is of great significance.

**Figure 1:**
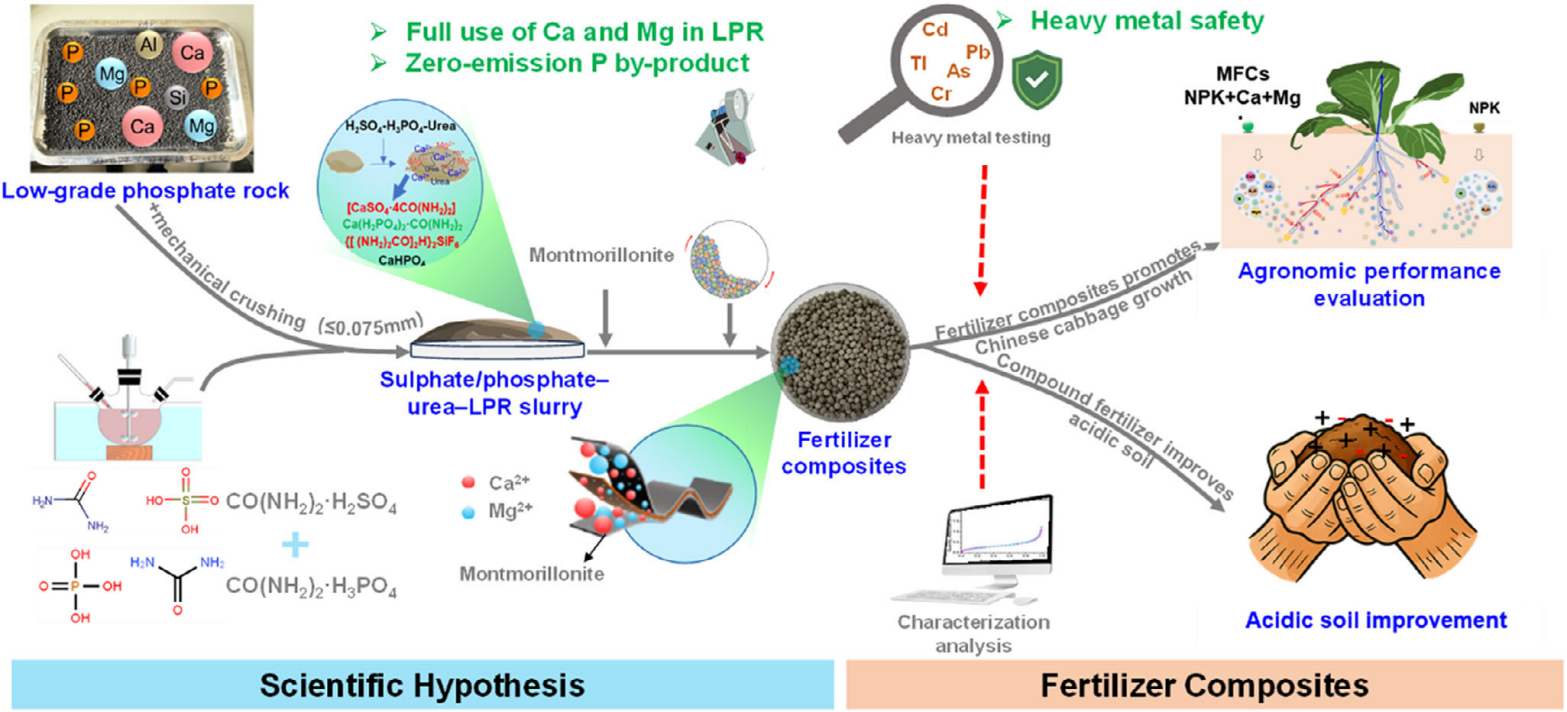
Scientific hypothesis, validation process, and the pathway from LPR to MFCs.

Intrinsically, the low activation efficiency of PR resources by strong acid is attributed to the fierce reaction, resulting in the coating of phosphogypsum onto the surface of PR and restricting its further reactions with strong acids. Reducing the acidity of reaction systems would be available practice to facilitate the resource utilization of PR using strong acids. Inspired by this, urea was introduced into H_2_SO_4_ activation system of PR for producing fertilizer composites, and interestingly, studies found that urea can effectively promote the progress of such a reaction.^[^ ^27^ ^]^ Nevertheless, urea‐induced H_2_SO_4_ activation still suffers from inadequate P, Ca, and Mg utilization. Besides, this process is limited by strict feedstock requirements, and the inherent mechanisms of nutrient release have not been comprehensively elucidated, thereby restricting its widespread application. Based on this, we emphasized the dominant role of acidity control in PR activation, and how to precisely control the acidity of reaction systems is still challenging. Given that PO_4_^3−^ is an inherent component of multifunctional fertilizer composites (MFCs), we ingeniously introduced PO_4_^3−^ (H_3_PO_4_) to achieve highly efficient activation via precisely controlling acidity and substantially improving the quality of MFCs (see details in **Figure** **1**). On the one hand, we speculated that introducing PO_4_^3−^ would enable precise control of the acidity, endowing this system with a synergistic acidolysis effect to break the crystal structures of PR. On the other hand, the production of citrate‐soluble nutrients (e.g., Ca(H_2_PO_4_)_2_) can endow MFCs with slow‐releasing properties. MFCs ameliorate acidic soil and alleviate Al toxicity stress by gradually releasing Ca and Mg.

Therefore, in this study, we systematically investigated the effect of PO_4_^3−^ on the acid‐activation of LPR and comprehensively elucidated the underlying mechanisms of nutrient activation and release, as well as its agricultural application. To the best of our knowledge, this is the first study to directly utilize LPR for the production of MFCs. These findings provide valuable technical and mechanistic insights into the zero‐waste utilization of LPR, and the as‐prepared MFCs possess significant potential for improving crop yield and ameliorating acidic soil.

## 2 Results and Analysis

### 2.1 Structural Characterization of P‐SUs

**Figure 2:**
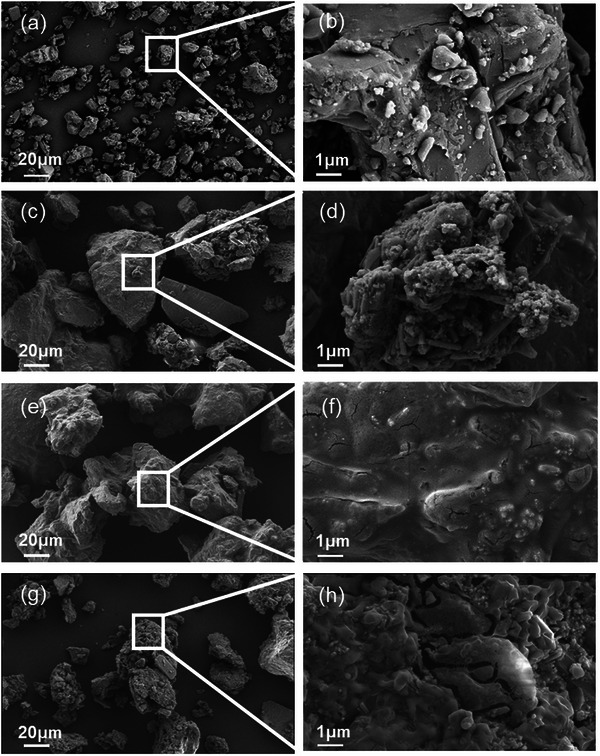
SEM images of activation acidolysis products in P‐SU0 a,b), P‐SU1 c,d), P‐SU2 e,f), and P‐SU3 g,h) at different magnifications.

Based on our hypothesis, the activation reactions between PSU and LPR involve the reaction of H^+^ with major LPR components, such as fluorapatite, CaCO_3_, and MgCO_3_. These reactions caused damage to the physicochemical structure of LPR and released a large amount of CO_2_, which consequently affected the morphological properties of acidolysis products. Scanning electron microscopy (SEM) images at different magnifications were obtained for the acidolysis products (**Figure** **2**). The morphology was significantly altered after PSU acid activation, and the PU ratio marked influence on the morphology of P‐SU1, P‐SU2, and P‐SU3. Specifically, compared to P‐SU0, the introduction of PO_4_^3−^ in P‐SU1, P‐SU2, and P‐SU3 resulted in rougher surfaces and larger pore size. In particular, P‐SU3 exhibited a coarse surface with numerous pore channels. The enhanced porosity and pore sizes suggest that PO_4_^3−^ modified the chemical composition of acidolysis products, and such a porous structure is likely to regulate nutrient release. A similar phenomenon has been reported in zeolite‐based nanocomposites, where a porous structure effectively moderates the release of nutrients.^[^ ^28^ ^]^

**Figure 3:**
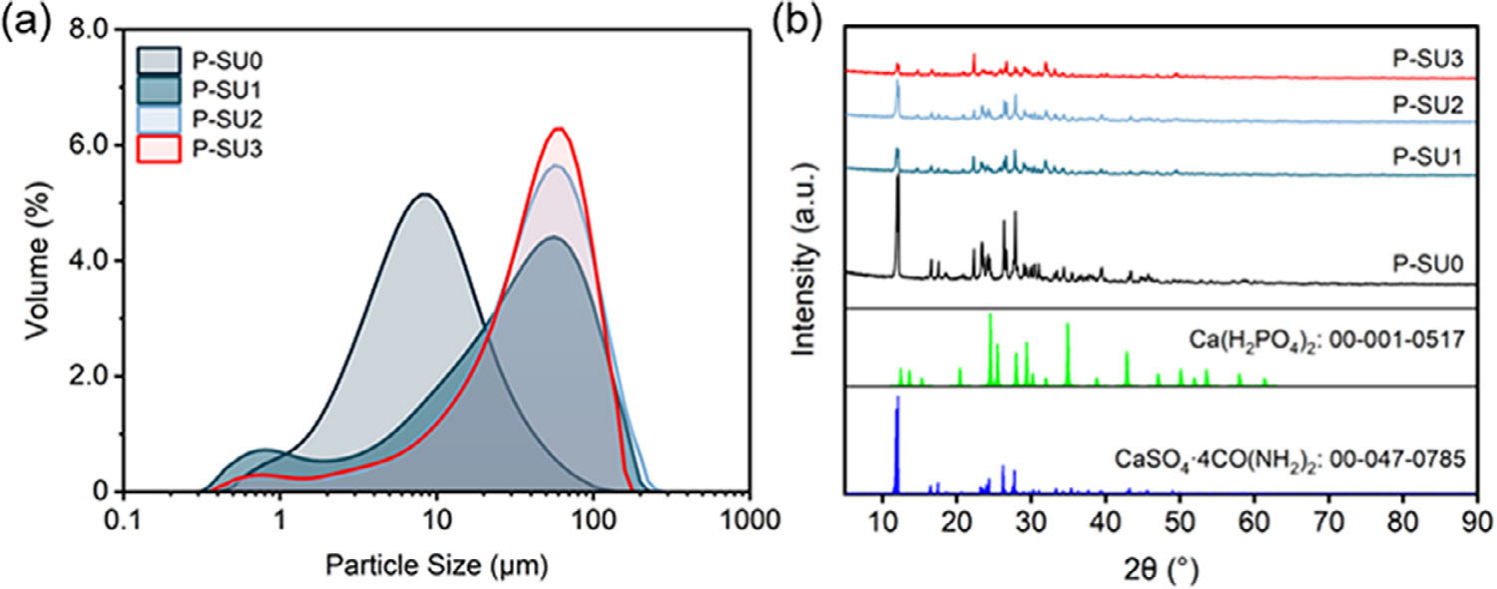
Particle size distribution a) and XRD patterns b) of activation acidolysis products in P‐SU0, P‐SU1, P‐SU2, and P‐SU3.

To further investigate study changes in particle size, the particle size distributions of the acidolysis products was measured (**Figure** **3**a). In contrast to the unimodal distribution of P‐SU0, the products P‐SU1, P‐SU2, and P‐SU3 exhibited bimodal distributions, with peaks in the ranges of 0.77–0.79 µm and 56.37–63.25 µm. As the PO_4_^3−^ addition increased, the particle size generally increased, which is consistent with the SEM observations (Figure S3, Supporting Information). Specifically, the D(90) values for P‐SU0, P‐SU1, and P‐SU2 were 24.17, 95.65, and 105.13 µm, respectively. Interestingly, the particle size of P‐SU3 decreased slightly compared to P‐SU2, with a D(90) of 92.88 µm (Table S2, Supporting Information). It is noteworthy that within the 0.3‐2.0 µm range, the intensity of the particle population in this specific range showed a significant enhancement with increasing PU addition. For P‐SU3, a slight reduction in particle size was observed, attributable to the inhibition of phosphogypsum crystallization by H_2_PO_4_^−^ derived from H_3_PO_4_‐urea (PU).^[^ ^29^ ^]^ X‐ray diffraction (XRD) analysis was performed to further investigate changes in the composition of the acidolysis products (Figure 3b). All samples showed clear peaks corresponding to CaSO_4_·4CO(NH_2_)_2_ and monocalcium phosphate Ca(H_2_PO_4_)_2_.^[^ ^30^ ^]^ Clear variations were found among the XRD patterns of the different acidolysis products (Figure 3b). In particular, the intensity of the CaSO_4_·4CO(NH_2_)_2_ peaks decreased in P‐SU1, P‐SU2, and P‐SU3 compared to P‐SU0, while that of Ca(H_2_PO_4_)_2_ increased. The primary reason for this is that a higher PO_4_^3−^ ratio promotes the formation of Ca(H_2_PO_4_)_2_.

### 2.2 Assessing Nutrient Activation of LPR by PSU

**Figure 4:**
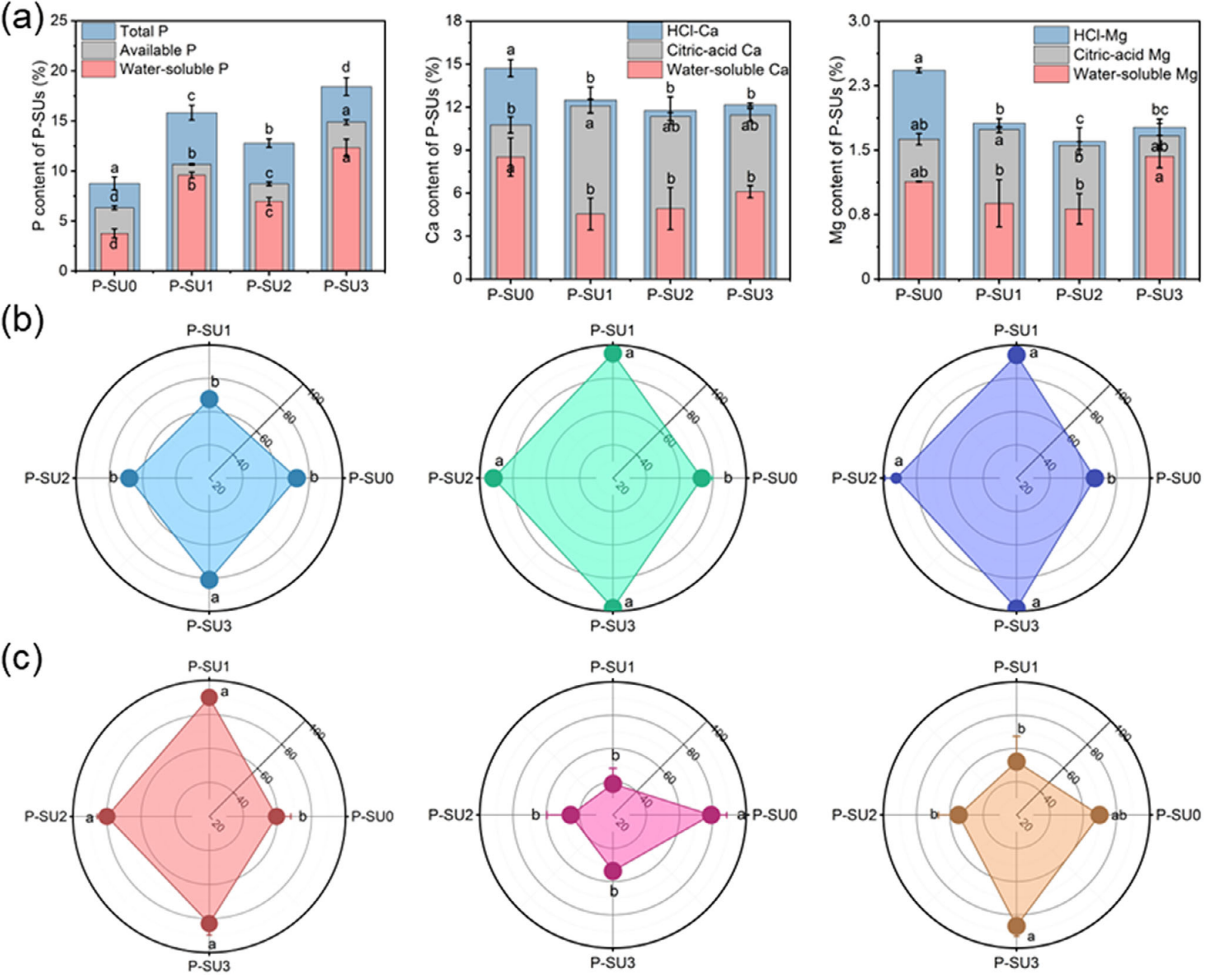
Content a), activation rates b), and ratio of water‐soluble to available nutrient c) of P, Ca, and Mg in the activation acidolysis products of P‐SU0, P‐SU1, P‐SU2, and P‐SU3. Data are presented as mean ± SD (*n* = 3). Significance was determined by one‐way ANOVA followed by Duncan's test. Different lowercase letters above the bars indicate significant differences among groups at *p* < 0.05.

Based on the structural characterization results, it was confirmed that the structure and composition of the acidolysis products were successfully altered, which subsequently affected nutrient activation performance. To validate this, the activation rates and the ratios of water‐soluble to available nutrients for the P‐SUs were determined (**Figure** **4**). The results demonstrated that as the proportion of PU in PSU system increased, the activation rates of P, Ca, and Mg, as well as the ratio of water‐soluble to available nutrients, exhibited significant improvement. Specifically, the activation rates increased from 72.5% to 81.1% for P, from 73.3% to 98.0% for Ca, from 67.0% to 98.3% for Mg. Regarding the ratio of water‐soluble nutrient to available nutrient, values increased from 59.5% to 90.0% for P, 69.8% to 86.7% for Mg. These findings are consistent with the structural characterization results; for example, the formation of Ca(H_2_PO_4_)_2_ as confirmed by XRD contributed to the increase in water‐soluble nutrients.

### 2.3 Nutrient Release Behaviors and Mechanisms of MFCs

**Figure 5:**
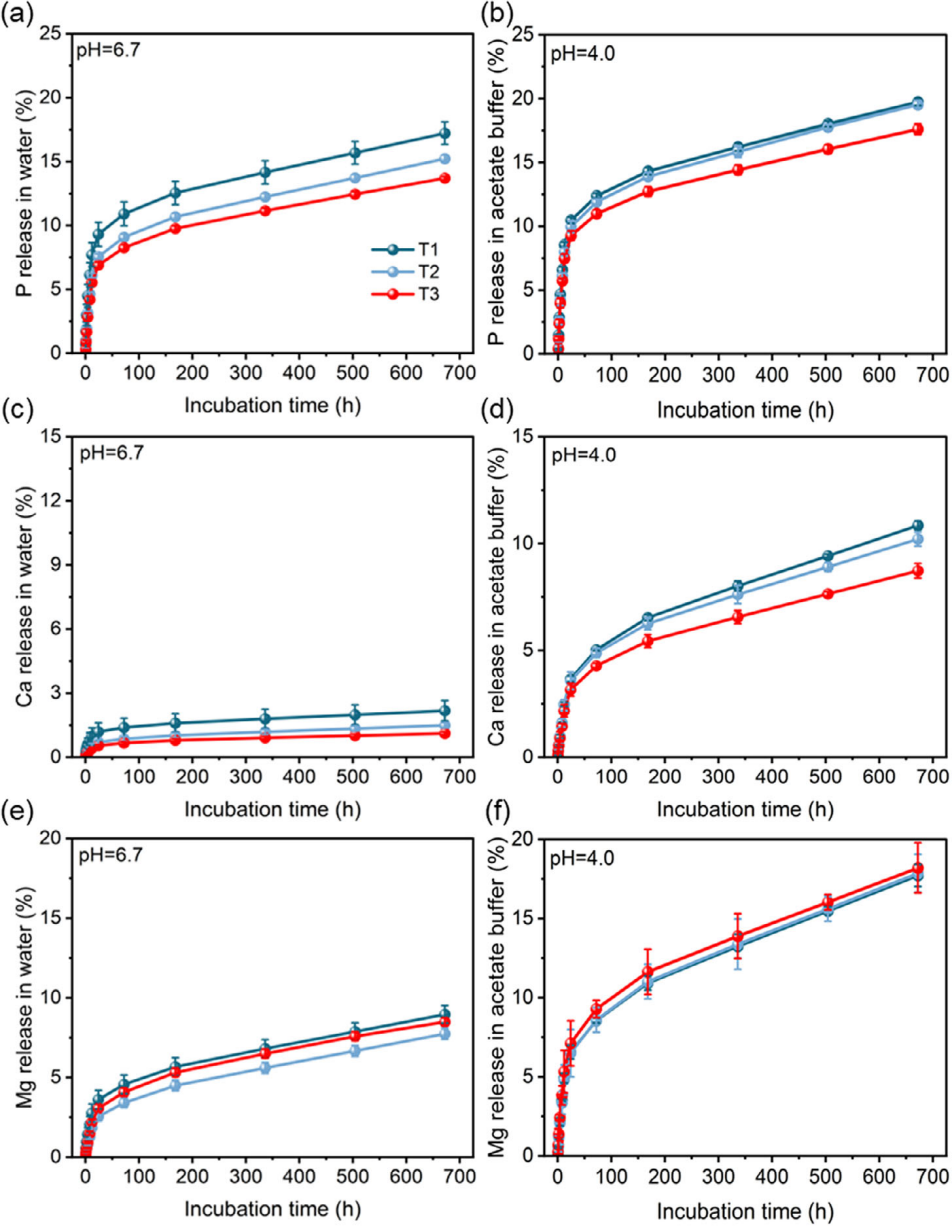
Release characteristics of P a,b), Ca c,d), and Mg e,f) from MFCs (T1, T2, and T3) under different pH conditions. Data are presented as mean ± SD (*n* = 3).

In general, the nutrient release characteristics of MFCs in solution and soil are critical for their practical application. Thus, a 28‐day nutrient release incubation experiment was conducted on the MFCs (T1, T2, and T3) under different pH conditions (**Figure** **5**). Overall, the cumulative nutrient release rates decreased in the order of T1 to T2 to T3, which can be attributed to the adsorption of nutrients by the porous structures. Notably, the solution pH significantly influenced nutrient release; as the pH decreased, the cumulative release rates increased. For T3, the cumulative release rates of P, Ca, and Mg at pH 6.7 and pH 4.0 were 13.7% and 17.6%, 1.1% and 8.7%, and 6.8% and 14.6%, respectively.

The nutrient release kinetics of the MFCs at pH 6.7 and 4.0 were simulated using the Korsmeyer‐Peppas model (Figure S4a–c, Supporting Information). The model fitting was considered robust, as evidenced by a high coefficient of determination (R^2^ > 0.80) coupled with low standard deviation for all fitted parameters (Figure S4d, Supporting Information). A decreasing trend in the constant k was observed from T1 to T2 to T3, indicating a reduction in the release rate of the MFCs. More importantly, the k values at pH 4.0 were lower than those at pH 6.7, suggesting that acidic conditions promoted nutrient release.

**Figure 6:**
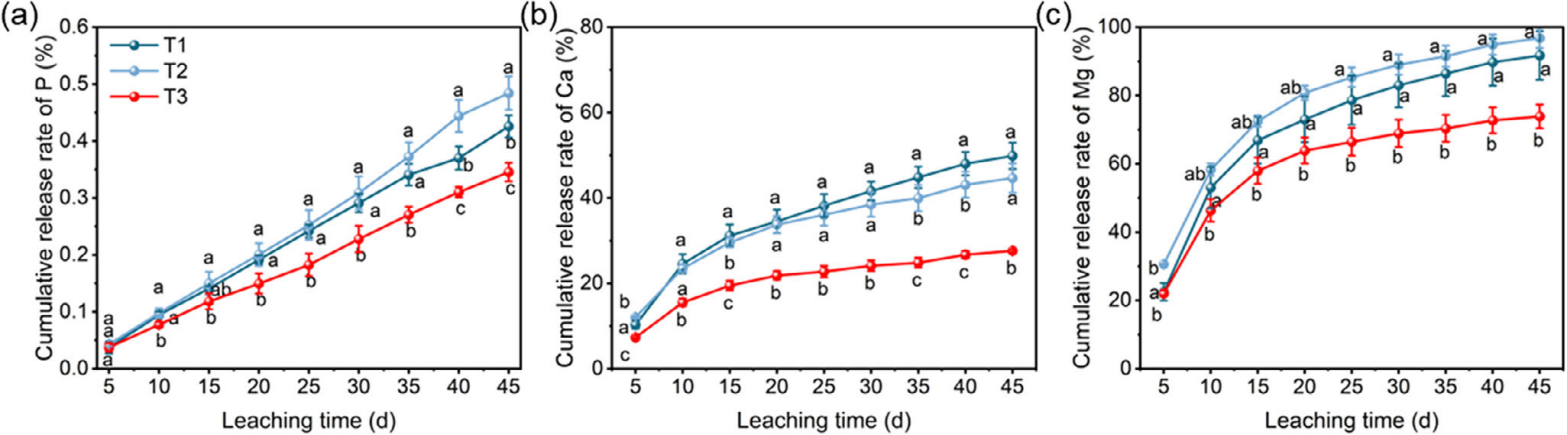
Cumulative leaching rates of P a), Ca b), and Mg c) from MFCs (T1, T2, and T3) in soil columns. Data are presented as mean ± SD (*n* = 3). Significance was determined by one‐way ANOVA followed by Duncan's test. Different lowercase letters above the data points indicate significant differences among groups at *p* < 0.05.

Furthermore, the nutrient release was evaluated through a 45‐day leaching experiment using acidic soil. Consistent with the solution‐based results, both the release amount and cumulative release rate of nutrients in soil decreased markedly from P‐SU1 to P‐SU2 to P‐SU3, particularly for Ca and Mg (**Figure** **6**; Figure S5, Supporting Information). The cumulative release rates of Ca and Mg for T1, T2, and T3 were 49.9%, 91.7%; 44.7%, 96.8%; and 27.6%, 73.9%, respectively. In contrast, P release was consistently slow across all treatments, which can be attributed to strong precipitation in acidic soil.^[^ ^31^ ^]^

**Figure 7:**
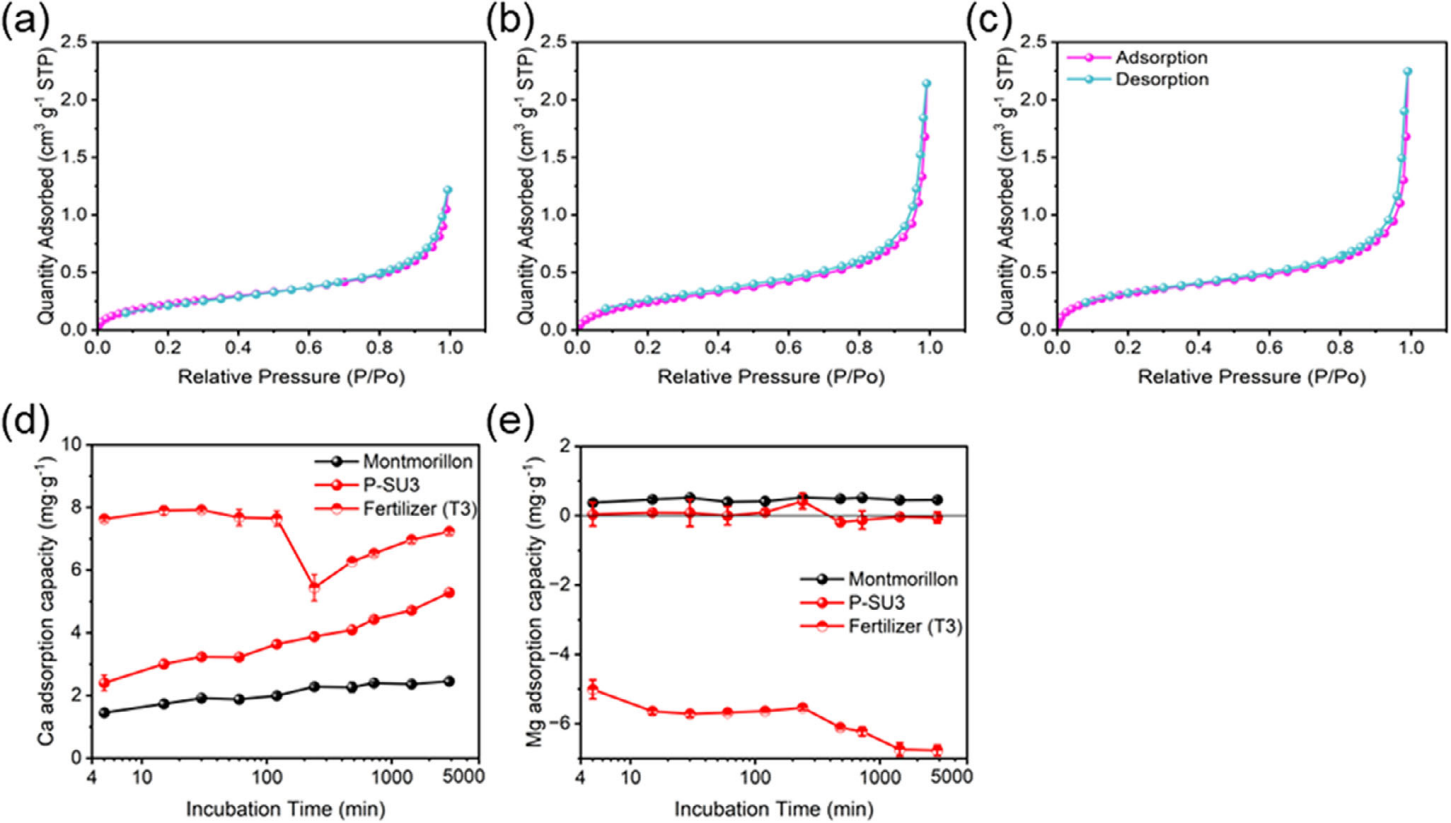
Role of pore structure in nutrient release from MFCs as revealed by BET analysis: a–c) Nitrogen adsorption‐desorption isotherms of T1, T2, and T3; d,e) Adsorption curves of Ca and Mg on montmorillonite, P‐SU3, and T3. Data are presented as mean ± SD (*n* = 3).

Finally, the nutrient release mechanism was elucidated through complementary characterization and experimental approaches. The porosity of the MFCs was investigated using N_2_ adsorption–desorption isotherms (**Figure** **7**). From T1 to T2 to T3, the MFCs exhibited a progressive increase in porosity. The specific surface area of MFCs increased from 0.88 to 1.20 m^2^ g^−1^, and a similar increasing trend was observed for pore volume (Table S3, Supporting Information). We speculated that such porous structures of MFCs may contribute to enhanced nutrient adsorption performance. To verify this, batch adsorption experiments were conducted for Ca^2+^ and Mg^2+^ using T3, with montmorillonite (a typical binder in fertilizer production) and P‐SU3 included for comparison (Figure 7d,e). The results revealed that T3 had a significantly higher adsorption capacity for Ca^2+^ than montmorillonite or P‐SU3 alone, indicating a synergistic effect between the components that promotes nutrient retention.

### 2.4 Multiple Agricultural Applications of MFCs

**Figure 8:**
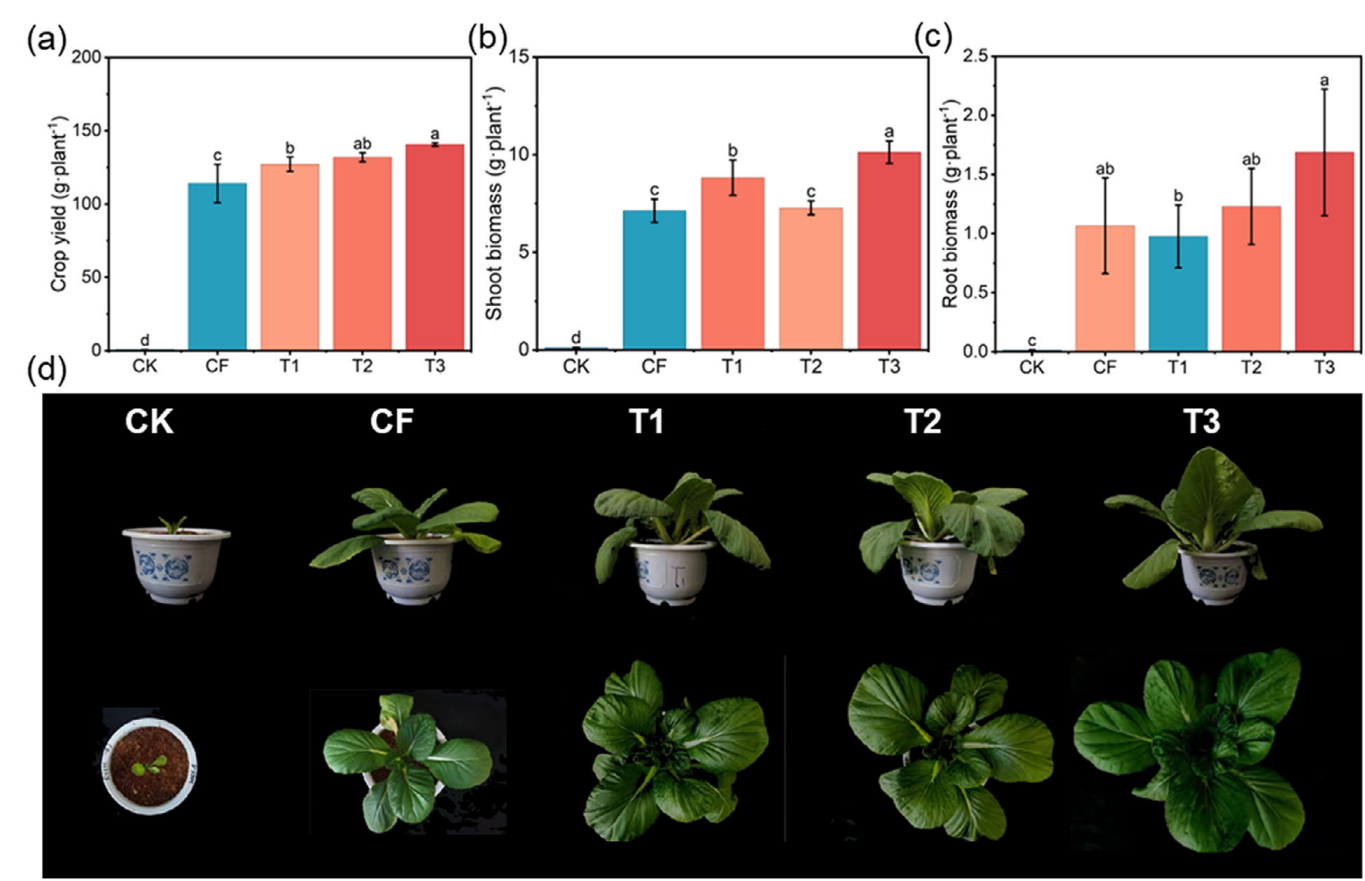
Crop yield a), shoot biomass b) and root biomass c) of Chinese cabbage in MFCs (T1, T2, and T3). Data are presented as mean ± SD (*n* = 3). Significance was determined by one‐way ANOVA followed by Duncan's test. Different lowercase letters above the bars indicate significant differences among groups at *p* < 0.05.

The agronomic performance of the MFCs was evaluated, and the results demonstrated that MFCs significantly enhanced the growth of Chinese cabbage, as evidenced by increased crop yield, shoot and root biomass, and SPAD values. Specifically, for T3, crop yield (**Figure** **8**a), shoot biomass (Figure 8b), root biomass (Figure 8c), root fresh weight (Figure S6, Supporting Information), and SPAD value (Figure S7, Supporting Information) increased by 23.4%, 42.0%, 18.2%, 43.7%, and 18.1%, respectively, compared to those of CF. These improvements could be attributed to the greater availability of Ca and Mg in the P‐SUs, which enhanced photosynthate synthesis and allocation in the shoots.

**Figure 9:**
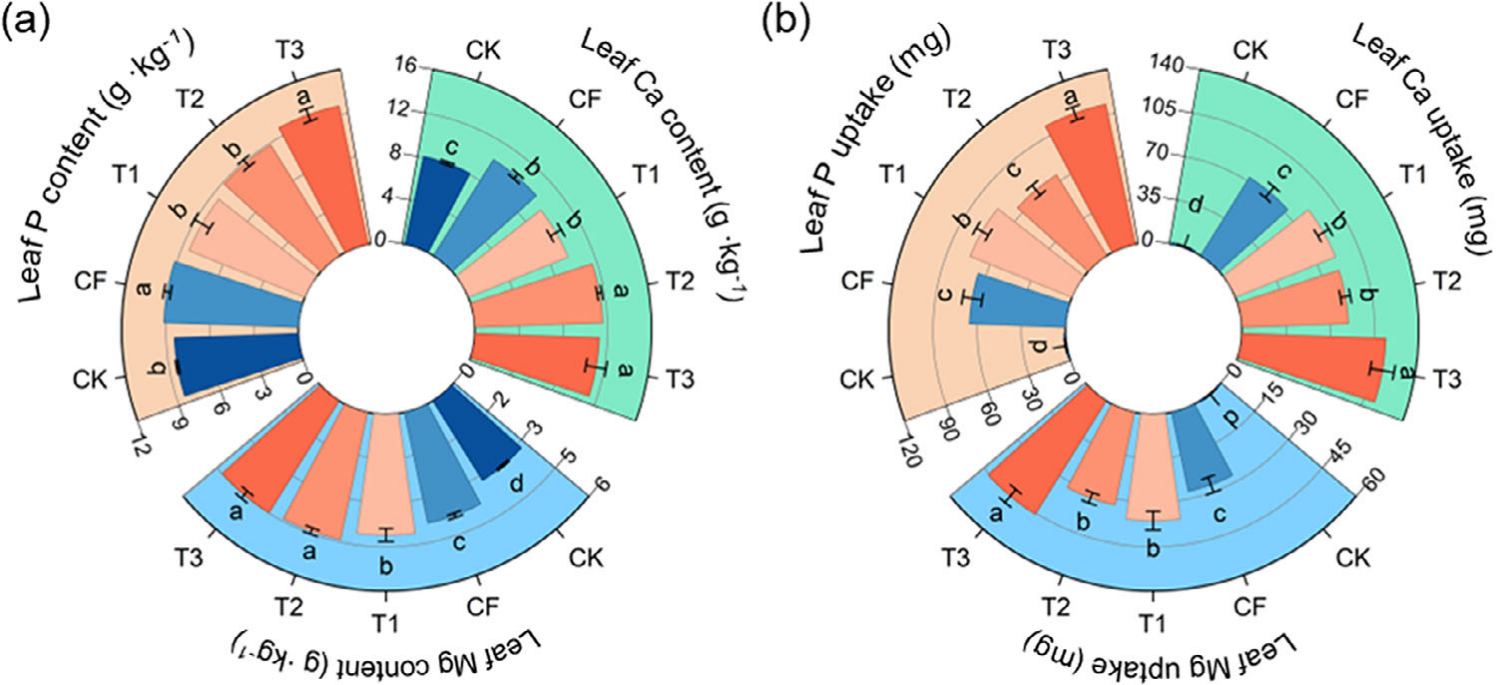
Leaf P, Ca, Mg content a) and uptake b) of Chinese cabbage in MFCs (T1, T2, and T3). Data are presented as mean ± SD (*n* = 3). Significance was determined by one‐way ANOVA followed by Duncan's test. Different lowercase letters above the bars indicate significant differences among groups at *p* < 0.05.

**Figure 10:**
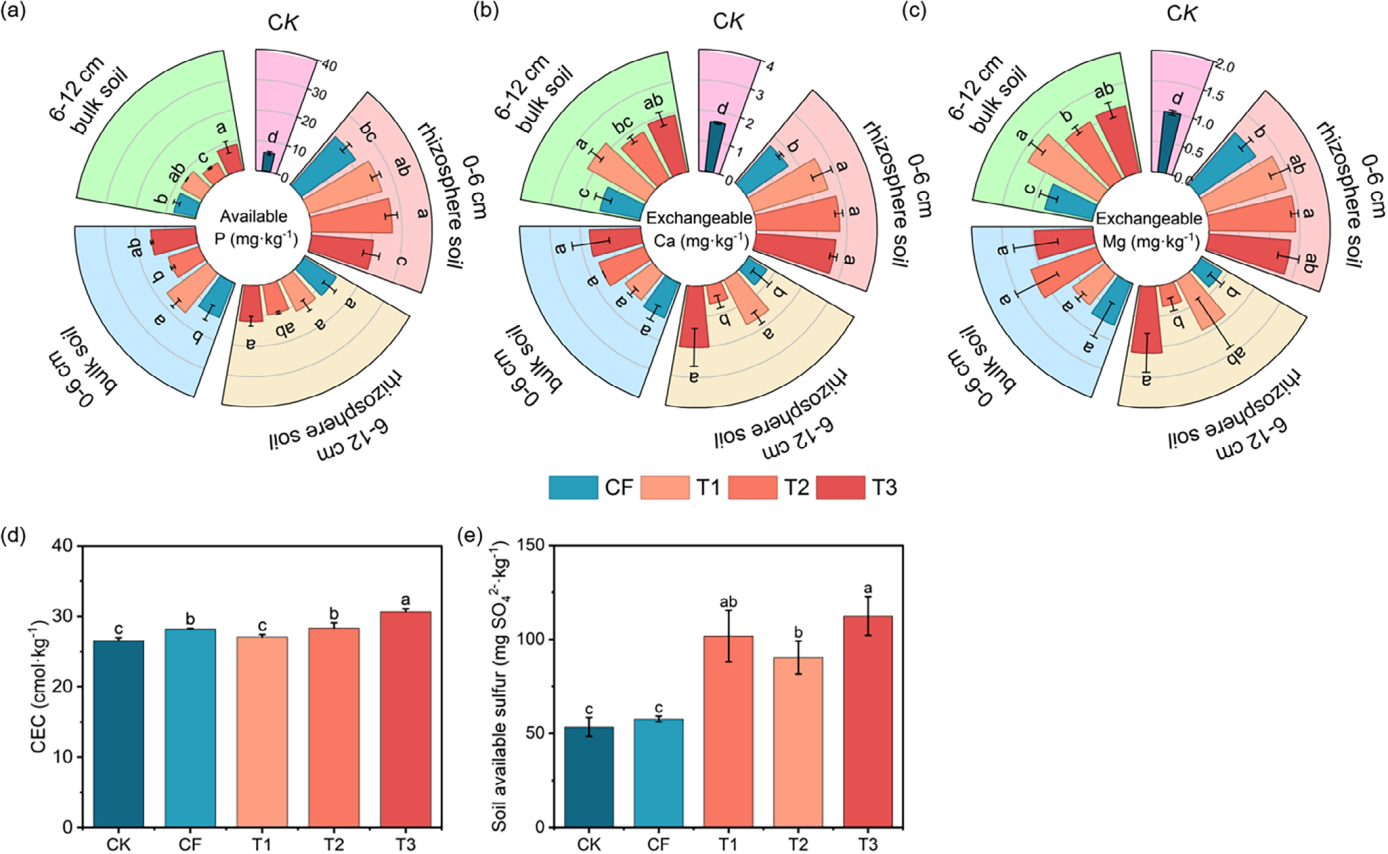
Soil nutrient content a–c) and acidic soil amelioration d,e) in MFCs (T1, T2, and T3). Data are presented as mean ± SD (*n* = 3). Significance was determined by one‐way ANOVA followed by Duncan's test. Different lowercase letters above the bars indicate significant differences among groups at *p* < 0.05.

Furthermore, the increased uptake of P, Ca, and Mg in plant leaves directly demonstrated the positive effects of the MFCs on nutrient assimilation. The leaf P, Ca, and Mg contents in plants treated with T3 showed significant increases of 4.9%, 10.0%, and 16.6%, respectively, compared to CF treatment (**Figure** **9**a). Similar trends were observed for the total uptake of P, Ca, and Mg in the leaves (Figure 9b). In addition, we evaluated the soil nutrient content, electrical conductivity (EC), and cation exchange capacity (CEC) in both rhizosphere and bulk soils at depths of 0–6 and 6–12 cm. The results indicated that the MFCs improved soil CEC (**Figure** **10**), EC (Figure S8, Supporting Information), and nutrient content, particularly the exchangeable Ca and Mg (Figure 10b,c), thereby enhancing nutrient retention and supply capacity.

**Figure 11:**
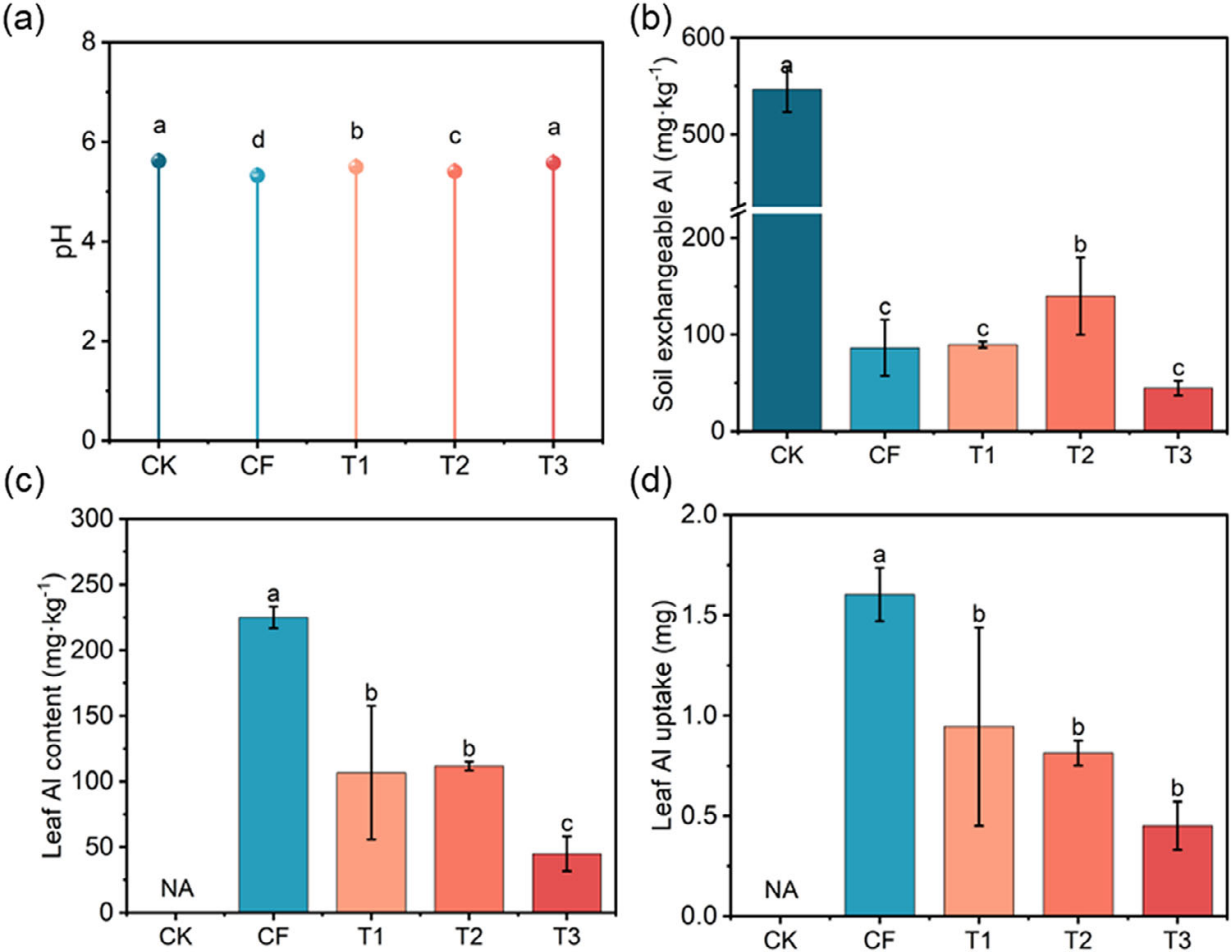
Soil pH a), soil Al availability b), and crop Al stress c,d) following the application of MFCs (T1, T2, and T3). NA: not assayed. No measurements were taken for the control (CK) group because of limited dry matter availability. Data are presented as mean ± SD (*n* = 3). Significance was determined by one‐way ANOVA followed by Duncan's test. Different lowercase letters above the bars indicate significant differences among groups at *p* < 0.05.

To investigate the potential of the MFCs for acidic soil amelioration, soil pH and exchangeable Al content were measured. The results indicated that the MFCs increased soil pH from 5.3 to 5.6 compared to CF treatment, while the effect on exchangeable Al content was either non‐significant or slightly increased (**Figure** **11**a,b). In addition, leaf Al content and Al uptake by plants decreased substantially by 52.6%–80.0% and 41.1%–71.9%, respectively, relative to CF (Figure 11c,d). The application of N‐containing fertilizers (e.g., urea, NH_4_^+^‐N) releases protons, resulting in soil acidification.^[^ ^32^ ^]^ MFCs effectively regulate the soil microenvironment through the slow release of Ca^2+^ and Mg^2+^. On one hand, MFCs buffer soil acidification (Figure 11a) and significantly reduce soil Al activity (Figure 11b). On the other hand, at the root‐soil interface, Ca^2+^ interacts with Al^3+^, forming a physicochemical barrier and competing for binding sites, thereby blocking Al uptake into plants.^[^ ^33^ ^]^ Meanwhile, Mg^2+^ enhances internal plant Al tolerance by competing for absorption sites, stimulating root organic acid secretion, and maintaining cellular ion homeostasis.^[^ ^34^, ^35^ ^]^ These coordinated mechanisms are expected to collectively reduce leaf Al content, potentially offering a viable strategy for mitigating Al stress in acidic soil.

Finally, to evaluate the ecological safety of the MFCs and their effects on soil and plant health, the contents of heavy metals (Cr, Cd, Pb, As, Hg, Tl) were determined. The results demonstrated no significant increase in heavy metal content in the MFCs, soil, or plants following agricultural application (Figures S9–S11, Supporting Information). All measured heavy metal contents were well below the limits stipulated by Chinese national standards (GB 38400‐2019, GB/T 15618‐2018, and GB 2762‐2022), confirming that the prepared MFCs are environmentally safe for agricultural use.

## 3 Discussion

### 3.1 Activation Mechanism of LPR via Acidity Control

The direct application of natural LPR is inherently limited due to the low solubility of crystalline P and poor synchrony with crop nutrient demand cycles.^[^ ^36^ ^]^ Industrially, activation of PR is predominantly achieved through decomposition with H_2_SO_4_.^[^ ^37^ ^]^ Previous studies have indicated that reducing the pH of the acidolysis system can enhance PR activation.^[^ ^38^ ^]^ However, an excessively low pH may induce a violent reaction leading to the formation of a CaSO_4_ coating, which can reduce activation efficiency.^[^ ^38^ ^]^ Moreover, this process requires an extended maturation period and involves high energy consumption. To address these challenges, this study developed an innovative acid‐regulating PSU activation process for LPR. The design of the PSU process fundamentally distinguishes it from conventional H_2_SO_4_ or H_3_PO_4_ decomposition routes such as those used in single superphosphate and triple superphosphate production. The incorporation of urea and H_3_PO_4_ significantly lowers the initial acidity of the H_2_SO_4_, moderating the reaction rates. This suppression of rapid reaction prevents the formation of a dense CaSO_4_ passivation layer,^[^ ^39^, ^40^ ^]^ thereby facilitating H^+^ diffusion and enhancing the activation efficiencies of P, Ca, and Mg in LPR (Figure 4). Further investigations revealed that the optimized ratio of the acidolysis agent results in mesoporous acidolysis products (Figure S12, Supporting Information). The abundant porous structures promote the interaction between H^+^ and LPR, thereby improving activation efficiency. This phenomenon has been demonstrated in prior work.^[^ ^38^, ^41^ ^]^ Moreover, the introduction of PO_4_^3−^ into the acidolysis agent increased the P_2_O_5_ content in the acidolysis products, enabling the direct preparation of MFCs while minimizing the generation of by‐products. This process, utilizing PSU activation of LPR, significantly enhances P, Ca, and Mg conversion efficiency, shortens the production process, enables full utilization of Ca and Mg, and reduces energy consumption. Notably, the process activates LPR via the PSU system, converting Ca and Mg from inert forms into plant‐available nutrients and effectively stabilizing them within the MFCs. This activation process fundamentally prevents the production of large volumes of solid waste rich in Ca and Mg, which is commonly generated in conventional acid‐based activation processes. Furthermore, it converts these elements into functional components that could ameliorate soil acidity and facilitate nutrient slow‐release. This achieves comprehensive resource utilization from element to product, showing simultaneous environmental and economic benefits. XRD analysis confirmed minimal batch‐to‐batch variation in the LPR used in this study (Figure S13, Supporting Information). To further establish the broader applicability of the proposed PSU process, future work should include validation using LPRs sourced from diverse geographical regions.

### 3.2 Nutrient Release of MFCs

P, Ca, and Mg are essential nutrients for plant growth, and their combined supply is critical for improving soil nutrient status and crop quality.^[^ ^42^, ^43^, ^44^ ^]^ However, conventional P fertilizers are predominantly produced from high‐grade PR (P_2_O_5_≥30%),^[^ ^45^ ^]^ which results in imbalanced Ca and Mg ratios and limits soil quality improvement. Moreover, the large surplus of P accumulated in soil (known as legacy P or residual P) can also be lost through leaching or runoff into freshwater ecosystems,^[^ ^46^ ^]^ causing nutrient pollution that threatens aquatic organisms.^[^ ^2^, ^47^ ^]^ Therefore, rational nutrient management in fertilizers is essential for enhancing nutrient availability in soil‐crop systems. In this study, the MFCs prepared via the PSU‐activated LPR process significantly reduced nutrient leaching in acidic soil (Figure 6). The pH‐dependent nutrient release behavior of the fertilizer (Figure 5) showed strong consistency with the soil column leaching tests, which could be attributed to two synergistic mechanisms. First, the optimized PU:SU ratio was critical for forming hierarchical pore structures during activation, as evidenced by the pore structure characterization (Figure S12, Supporting Information). This well‐developed porosity facilitates gradual nutrient diffusion (Figure 7), thereby enhancing the slow‐release properties. Second, the incorporation of montmorillonite establishes a cation–exchange–regulated release system with high cation exchange capacity,^[^ ^48^ ^]^ which enables selective retention of Ca^2+^/Mg^2+^ and effectively mitigates leaching losses (Figure 6). To further validate this mechanism, we conducted pore structure characterization and Ca^2+^/Mg^2+^ adsorption experiments on the MFCs (Figure 7d,e). The results confirm that the fertilizer derived from PSU‐activated LPR possesses a well‐developed porous structure and exhibits strong adsorption capacity for Ca^2+^. The relationship between pore properties and the fitted nutrient release parameters was further analyzed, and a consistent trend between meso‐pore and k value was found, particularly in T2 and T3 (Figure S14, Supporting Information). This observation is consistent with previous reports on nutrient slow‐release from porous materials.^[^ ^28^, ^49^ ^]^

### 3.3 Multifunctional Effects on Crop Growth and Acidic Soil Amelioration

The soil available P (AP) content serves as a key indicator of soil P supply capacity,^[^ ^19^ ^]^ providing direct guidance for fertilization practices. Previous studies have demonstrated that activated fertilizer composites produced through various processes can enhance soil AP content and increase crop biomass.^[^ ^36^, ^50^, ^51^ ^]^ In this study, we employed a PSU‐activated LPR process to fabricate MFCs that not only meet crop P demands throughout the growth cycle but also effectively activate Ca/Mg nutrients from LPR (Figure 4). Unlike conventional fertilizers, where P, Ca, and Mg are prone to loss,^[^ ^52^, ^53^, ^54^, ^55^ ^]^ MFCs possess slow‐release properties that not only mitigate the environmental pollution risk from P^[^ ^52^, ^56^ ^]^ but also significantly reduce the leaching loss of Ca and Mg. The resulting slow‐release behavior of these essential nutrients (Figures 5 and 6) establishes a continuous nutrient supply mechanism in soil, facilitating efficient crop uptake and utilization (Figures 8 and 9; Table S4, Supporting Information). Moreover, the efficient nutrient supply system of these composites shows significant potential for acidic soil amelioration.

Acidic soil account for 40‐50% of the world's potentially arable land.^[^ ^57^ ^]^ In China, ≈20 million hectares of acidic soil (pH < 6.5) have been identified, which were predominantly distributed across southern regions.^[^ ^51^ ^]^ These soils exhibit elevated levels of Fe and Al oxides with high chemical reactivity. Under highly acidic conditions (pH<5.5), these oxides readily bind with P to form insoluble Fe‐P and Al‐P complexes, thereby reducing the availability of soil AP and alkaline cations (Ca^2+^, Mg^2+^, K^+^), while simultaneously exacerbating Al toxicity in plants.^[^ ^57^ ^]^ These constraints ultimately limit crop productivity.^[^ ^58^, ^59^ ^]^ Conventional inorganic amendments for acidic soil remediation primarily include lime‐based materials (e.g., quicklime and hydrated lime) and mineral conditioners (e.g., oyster shell powder, fly ash, alkaline slag, dolomite, phosphogypsum, and PR).^[^ ^58^, ^59^, ^60^, ^61^ ^]^ Lime materials rapidly raise the soil pH by neutralizing exchangeable H^+^ and Al^3+^ while simultaneously supplementing Ca nutrients and mitigating Al toxicity.^[^ ^59^, ^62^, ^63^, ^64^ ^]^ In contrast, mineral conditioners gradually improve soil structure and enhance P availability through slow‐release mechanisms. However, prolonged and excessive lime application can lead to soil compaction and secondary acidification,^[^ ^65^ ^]^ whereas mineral conditioners often exhibit inefficient nutrient release kinetics and provide insufficient Ca/Mg supplementation. In this study, we fabricated MFCs through the PSU‐activation LPR process. The synergistic interaction between montmorillonite and acidolysis products establishes a dual release system for P, Ca, and Mg (Figure 6). The gradual nutrients release, particularly Ca and Mg, effectively enriches soil nutrient content (Figure 10a–c), buffers soil acidification induced by fertilization (Figure 11a), suppresses Al activity (Figure 11b), and alleviates Al toxicity in plants (Figure 11c,d). This innovative P‐Ca‐Mg synergistic system resolves the longstanding limitation of traditional amendments, which often either raise pH without supplying sufficient P, or supply P without effectively stabilizing soil pH.

PR may contain contaminants, such as Cd, which can transfer into fertilizer products, accumulate in soils, and eventually enter the food chain.^[^ ^66^, ^67^ ^]^ This study systematically validates an integrated safety framework for heavy metals, encompassing: 1) risk‐controlled synthesis of MFCs from LPR (Figure S9, Supporting Information), 2) environmentally benign field application (Figure S10, Supporting Information), and 3) the production of safe agricultural products (Figure S11, Supporting Information). This framework establishes scientific principles and technical protocols for the eco‐efficient utilization of mineral resources and sustainable agricultural intensification. To facilitate the field‐scale application of MFCs, future work needs to be focused on the following key aspects. First, a systematic environmental safety assessment must be performed, including comprehensive screening for a full panel of potentially toxic elements and persistent substances. Second, long‐term field trials must be conducted based on standards or regulations established by China or the European Union, to establish the field‐scale application of MFCs across diverse soil types and crop species.

### 3.4 Industry and Agriculture Implications

**Figure 12:**
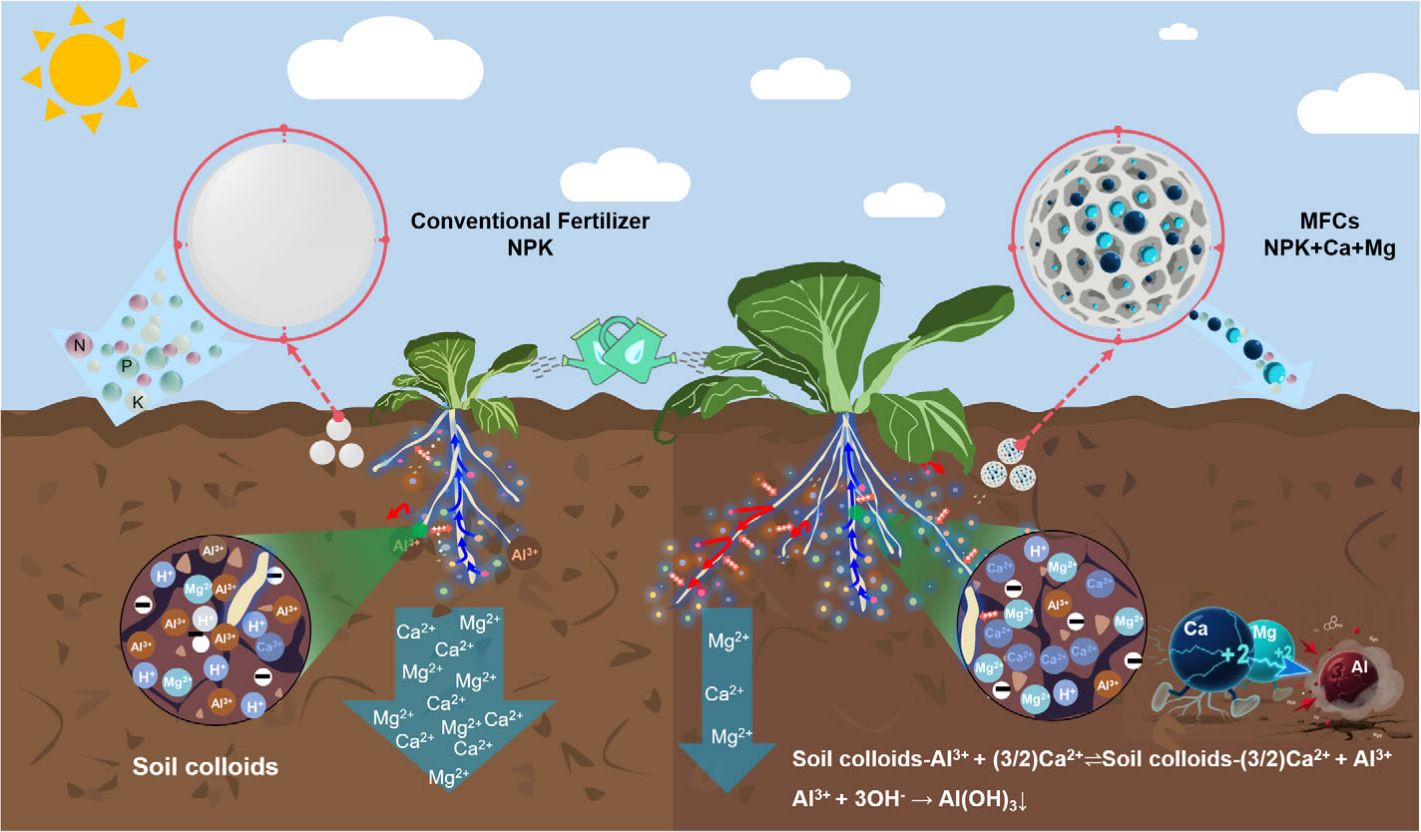
A synergistic system for sustainable agriculture established by MFCs application, integrating nutrient slow‐release, acidic soil amelioration, root nutrient acquisition, and crop yield enhancement.

Global demand for PR is increasing rapidly, while the industry faces the dual challenges of depletion of high‐grade PR resources and the environmentally detrimental processing of low‐grade deposits, particularly in China. This study presents a strategy for the direct preparation of MCFs from LPR, which integrates slow‐release nutrient supply, acidic soil amelioration, and environmental compatibility. The developed approach effectively minimizes the generation of P by‐products typically associated with conventional flotation and wet‐process H_3_PO_4_ technologies. Furthermore, preliminary calculations indicate that under the applied process conditions, the production of each ton of the MFCs (T3) reduces emissions of phosphorus tailings and phosphogypsum by 18.87% and 19.98%, respectively, compared to CF (Table S5, Supporting Information). Through the gradual release of Ca and Mg, the MCFs can alleviate soil acidification, immobilize exchangeable Al, enhance soil ECE, and reduce leaching losses of base cations. These improvements collectively promote root development and nutrient acquisition in Chinese cabbage, ultimately establishing a clear causal pathway from nutrient slow‐release through acidic soil amelioration and rhizosphere modification to significantly increased crop yield (**Figure** **12**).

While current research has successfully established a multifunctional nutrient cycling system that integrates slow‐release fertilizer composites with acidic soil amelioration to enhance efficient nutrient uptake and utilization, future studies will focus more on the role of soil microorganisms within this system. Notably, the predominant citrate‐soluble nutrient form intrinsically limits the fertilizer's applicability to acidic soil. Comprehensive validation through systematic field trials across diverse soil types and crop species remains essential to fully assess its agricultural potential, environmental benefits, and economic viability.

## 4 Conclusion

The full utilization of LPR remains highly challenging, and conventional phosphate fertilizer production generally generate substantial waste. Herein, we fabricated MFCs through the novelly designed PSU‐activation LPR process and evaluated their agronomic performance. The results indicated that under optimized conditions, the activation efficiencies of P, Ca, and Mg reached 78.5%–98.3%. The resulting porous structure enhanced adsorption capacity and slow‐release performance, reducing the leaching rates of Ca and Mg to 27.6% and 73.9%, respectively. Pot experiments demonstrated that the MFCs significantly increased the biomass (by 11.5%–23.4%) and enhanced the leaf P, Ca, and Mg contents (by 4.9%–16.6%) of Chinese cabbage compared to CF. Furthermore, MFCs were proven to be environmentally friendly, showing no significant risk of heavy metal contamination in soil or crops. In conclusion, these findings provide novel insights into the direct utilization of LPR for fertilizer production, offering economic, environmental, and agronomic benefits. Future research need to focus on large‐scale field trials to comprehensively evaluate the environmental impact and resource use efficiency of this technology across diverse soil‐crop systems.

## 5 Experimental Section

### Chemical Reagents and Materials

The LPR with 19.0 wt% P_2_O_5_, sourced from Yunnan Yuntianhua Co., Ltd., Kunming, China, as a representative sample, was pretreated by planetary ball milling to achieve particle sizes below 75 µm for subsequent experiments. The contents of MgO, CaO, and Al_2_O_3_ were 7.59 wt.%, 38.5 wt.%, and 1.5 wt.%, respectively, along with an ignition loss of 13.6 wt% (950 °C, 30 min). The heavy metal content of LPR is presented in Table S1 (Supporting Information). All chemical reagents were used as received without further purification. H_3_PO_4_ (≥85 wt.%), H_2_SO_4_ (≥98 wt.%), hydrochloric acid (HCl, 36–38 wt.%), citric acid, acetone, sodium dodecyl sulfate (SDS, C_12_H_25_NaO_4_S), disodium ethylenediaminetetraacetate (EDTA, ≥99 wt.%), and quinoline were of analytical grade. Urea (N ≥46 wt.%), diammonium phosphate (DAP, (NH_4_)_2_HPO_4_, N ≥18 wt.%, P_2_O_5_ ≥46 wt.%), and potassium chloride (KCl, K_2_O ≥60 wt.%) were provided by Yunnan Yuntianhua Co., Ltd., Kunming, China. Montmorillonite, as granulating binder, was purchased from Ruixin Mineral Powder Factory in Lingshou County, China.

### Preparation of Acidolysis Products using PSU

H_3_PO_4_‐urea (PU) solution was prepared by reacting H_3_PO_4_ and urea at a molar ratio of 1:0.8 at 78 °C with stirring at 300 rpm for 30 min. Under similar conditions, H_2_SO_4_‐urea (SU) solution was prepared using H_2_SO_4_, urea, and water at a molar ratio of 1:3.6:1. In order to precisely control the acidity, binary mixtures of PU and SU (PSU) were prepared at volume ratios of 0:3, 1:2, 1:1, and 2:1, denoted as PSU0, PSU1, PSU2, and PSU3, respectively. The volume of PSU solution added to 50 g of LPR was 105% of the theoretical amount required for complete activation of the LPR. The SDS‐urea solution was prepared by thoroughly mixing SDS, urea, and water in a beaker at room temperature (25 ± 2 °C).

The experimental procedures for preparing acidolysis products were shown as follows: the SDS‐urea solution was added to a specified quantity of LPR, and then stirred continuously to produce SDS‐urea‐LPR slurry. PSU0, PSU1, PSU2, and PSU3 solutions were added slowly to the SDS‐urea‐LPR slurry individually in the reactor at 80 °C, and the reactions were carried out under stirring at 400 rpm for 30 min. After reaction, the acidolysis products were frozen at −80 °C for 1 h to quench the reaction and then thawed to room temperature prior to characterization. The resulting acidolysis products were labelled P‐SU0, P‐SU1, P‐SU2, and P‐SU3, respectively (Figure S1, Supporting Information).

The structural properties of the acidolysis products were systematically characterized. Morphological features were visualized using SEM (Zeiss Sigma300, Carl Zeiss AG, Germany). Prior to SEM imaging, a thin layer of gold was sputter‐coated onto the sample surface for 120 s to enhance conductivity and minimize charging effects. The crystalline phase composition was determined by XRD (Ultima IV, Rigaku, Japan). In detail, a scanning range of 5–90° with a 2θ scanning speed of 10°·min^−1^ was adopted, and the XRD patterns were recorded using MDI Jade software (Version 6.5, Materials Data, Inc., USA). Particle size distributions were determined using a laser diffraction particle size analyzer (Mastersizer 2000, Malvern Panalytical, UK).

The activation efficiency of PSU in activating LPR was evaluated based on activation rates (*r*, %) and the ratio of water‐soluble nutrient to available nutrient (*w*, %) for P, Ca, and Mg in P‐SUs, according to the following equations:^[^ ^19^ ^]^

(1) $$\begin{array}{ccc} & & r_{P}=\frac{\gamma_{P}}{\beta_{P}}\times 100\%,\;r_{\mathrm{Ca}}=\frac{\gamma_{\mathrm{Ca}}}{\beta_{\mathrm{Ca}}}\times 100\%,\;r_{\mathrm{Mg}}=\frac{\gamma_{\mathrm{Mg}}}{\beta_{\mathrm{Mg}}}\times 100\%,\\ & & w_{P}=\frac{\alpha_{P}}{\gamma_{P}}\times 100\%,\;w_{Ca}=\frac{\alpha_{Ca}}{\gamma_{Ca}}\times 100\%,\;\mathrm{and}\;w_{\mathrm{Mg}}=\frac{\alpha_{Mg}}{\gamma_{Mg}}\times 100\% \end{array}$$

where *α*_P_, *γ*_P_, *β*_P_, *α*_Ca_, *γ*_Ca_, *β*_Ca_, *α*_Mg_, *γ*_Mg_, and *β*_Mg_ are the water‐soluble P, available P, total P, water‐soluble Ca, citric acid‐soluble Ca, hydrochloric acid‐soluble Ca, water‐soluble Mg, citric acid‐soluble Mg, and hydrochloric acid‐soluble Mg of the P‐SUs, respectively. The concentrations of *α*_P_, *γ*_P_, and *β*_P_ were quantified by distilled water, 37.5 g L^−1^ EDTA solution, and aqua regia (HCl:HNO_3_ = 3:1, v:v), respectively. The concentrations of *α*_Ca_/*α*_Mg_, *γ*_Ca_ /*γ*_Mg_, and *β*_Ca_/*β*_Mg_ were quantified by distilled water, 20 g L^−1^ citric acid solution, and HCl solution (HCl:H_2_O = 1:1, v:v), respectively. The concentrations of Ca and Mg in the extracts were determined using inductively coupled plasma optical emission spectrometry (ICP‐OES, Agilent 5110, Agilent Technologies, USA).

### Preparation of MFCs and its Comprehensive Evaluation

*Preparation procedures*: Using the acidolysis products, we prepared MFCs through the following procedure: ammonia (NH_3_) was introduced into the P‐SUs to adjust the pH of 6.5–7.0, resulting in ammoniation of the acidolysis products. The ammoniated products are denoted as P‐SUAs. To evaluate the agronomic effects, Chinese cabbage (a typical crop) was selected. The MFCs were granulated using the P‐SUAs, DAP, KCl, and urea in varying proportions, to achieve a target nutrient ratio of N:P_2_O_5_:K_2_O = 17:10:11. To ensure consistent P content across all experimental treatments, the MFCs were designed as follows: a 6:4 mass ratio of P‐SUA1 to DAP, a 5:5 mass ratio of P‐SUA2 to DAP, and used P‐SUA3 as the sole P source, designated as T1, T2, and T3, respectively.

*The nutrient release behaviors and mechanisms*: Based on experimental and analytical methods, the nutrient release behaviors and underlying mechanisms were investigated. The release pattern of P, Ca, and Mg was examined in different pH buffer solutions and through soil column experiments. The nutrient release characteristics of the MFCs (T1, T2, and T3) were evaluated by incubating samples in two solutions (deionized water (pH = 6.7) and acidic aqueous solution (pH = 4.0)) for 672 h at 25 ± 2 °C with shaking at 100 rpm. Solution samples were collected at 0.5, 1, 2, 4, 8, 12, 24, 72, 168, 336, 504, and 672 h. At each time point, 5 mL of supernatant was withdrawn after allowing 5 min of settling, and an equal volume of fresh solution was replenished. Cumulative release rates (δ, %) of P, Ca, and Mg in MFCs were calculated using the following equation:

(2) $$\delta =\frac{\sum_{i=1}^{n}(Ci\times V_{\mathrm{sample}})}{W_{\mathrm{total}}}\times 100\%$$

where *C_i_* is the concentration (mg L^−1^) of the P, Ca, or Mg in the sample; *V*_sample_ is the constant sample volume (5 mL); and *W*_total_ is the total mass (mg) of the nutrient in MFCs. The Korsmeyer‐Peppas model was employed to evaluate nutrient release characteristics of MFCs under different pH conditions.^[^ ^68^ ^]^ The fitting equation is expressed as: $\frac{Q_{t}}{Q_{\max}}$ = *kt^n^*, where *Q_t_* (mg) is the cumulative release amount at time t; *Q*_max_ (mg) is maximum release amount; *k* is release rate constant; *n* is release exponent.

A column leaching test was conducted using polyvinyl chloride pipes (12 cm in diameter, 35 cm in length) filled with acidic soil to evaluate nutrient leaching characteristics of MFCs. Soil was collected from Kunming, Yunnan, China (102°30′E, 24°49′N), and its physicochemical properties were determined: pH 5.7, organic matter 56.6 g kg^−1^, total N 0.8 g kg^−1^, available P 1.1 mg kg^−1^, available K 724 mg kg^−1^, exchangeable Ca 11.8 cmol kg^−1^, and exchangeable Mg 7.4 cmol kg^−1^. To improve soil aeration, coir was mixed with the experimental soil at a 1:2 mass ratio. Based on the typical growth period of Chinese cabbage, leaching was carried out over 45 days with a total of 1900 mL deionized water applied in increments of 211.1 mL per leaching event. Leachate was collected every 5 days and analyzed for water‐soluble P, Ca, and Mg.

The nutrient release mechanisms were further elucidated through adsorption experiments and material characterization. For the adsorption kinetics, the adsorption of Ca^2+^ and Mg^2+^ on montmorillonite (as a reference), P‐SU3, and T3 was investigated in a mixed‐ion solution (100 mg L^−1^ Ca^2+^ and 50 mg L^−1^ Mg^2+^) under shaking (25 ± 2 °C, 150 rpm). Samples were collected after 5, 15, and 30 min, and 1, 2, 4, 8, 12, 24, and 48 h. Following centrifugation and filtration (0.45 µm), the residual Ca^2+^ and Mg^2+^ concentrations were measured by ICP‐OES, and the adsorption capacities (*q_t_*, mg g^−1^) were calculated according to:^[^ ^18^ ^]^

(3) $$q_{t}=\frac{\left(C_{0}-C_{t}\right)\times V}{m}$$

where *C*_0_ (mg L^−1^) is the initial concentration; *C*_t_ (mg L^−1^) is the solution concentration at time t; *V* (L) is the volume; *m* (mg) is the mass of the adsorbent. For the latter, the porous properties of the MFCs were analyzed using N_2_ adsorption‐desorption isotherms. Prior to measurement, T1, T2, and T3 were dried at 105 °C for 12 h under vacuum. The N_2_ isotherms were obtained using a Micromeritics ASAP 2460 (Version 4.03, Micromeritics Instrument, USA). The *t*‐plot method was employed to determine the microporosities (< 2 nm).

### Multiple Agricultural Applications of MFCs

To assess the effects of the MFCs on crop growth and soil amelioration under acidic conditions, a 45‐day pot experiment using Chinese cabbage (*Brassica rapa L. ssp. Pekinensis “*KY28*”*) was conducted. The experiment included five treatments with five replicates each: CK (no fertilizers), CF (conventional fertilizer with an N‐P_2_O_5_‐K_2_O ratio of 17‐10‐11), and T1, T2, and T3. The soil used was identical to that described in the leaching experiment. Each pot was filled with 1.6 kg of air‐dried soil and amended with the corresponding MFCs at application rates equivalent to 300 mg N, 176 mg P_2_O_5_, and 190 mg K_2_O per kg of soil. Four Chinese cabbage seeds were sown per pot, and thinned to one plant per pot at the two‐leaf stage. The experiment was conducted in a controlled‐environment growth chamber under a diurnal temperature regime of 28 °C (14 h day) and 15 °C (10 h night). After 45 days of growth, the following parameters were measured: root biomass, shoot biomass, leaf SPAD (Soil and Plant Analyzer Development) value, and the concentration and uptake of nutrients (P, Ca, and Mg).

To assess the effects of the MFCs on soil physicochemical properties and their efficiency in ameliorating soil acidity, a stratified sampling protocol was implemented at maturity. The potted soil system was divided into two zones at depths of 0–6 cm and 6–12 cm, and then bulk soil and rhizosphere soil were collected (Figure S2, Supporting Information). Soil samples were analyzed for pH, EC, CEC, available P, exchangeable cations (Ca^2+^, Mg^2+^, and Al^3+^).

To further evaluate environmental and ecological safety, heavy metal concentrations (Cr, Cd, Pb, As, Hg, and Tl) in the LPR, MFCs, soil, and crop tissues were determined in accordance with Chinese national standards (GB 38400‐2019, GB/T 15618‐2018, and GB 2762‐2022).

### Statistical Analysis

In current study, all treatments were performed in triplicate, and data are presented as mean ± standard deviation (SD). Statistical analysis was performed using SPSS (version 22.0, IBM, USA). Differences among treatments were analyzed by one‐way ANOVA, followed by Duncan's post‐hoc test for multiple comparisons at a significance level of *p* < 0.05.

## Conflict of Interest

The authors declare no conflict of interest.

## Supporting information



Supporting Information

## Data Availability

Research data are not shared.
