# Correction to “Multifunctional Tailoring of Fertilizer Composites Directly Derived From Phosphate Rock”

**DOI:** 10.1002/advs.76404

**Published:** 2026-07-03

Z. Qi, J. Wang, L. Chen, et al. “Multifunctional Tailoring of Fertilizer Composites Directly Derived From Phosphate Rock.” *Adv. Sci*. 13, no. 11 (2026): e17533. https://doi.org/10.1002/advs.202517533

In Figure 4a, the significance markers for the total P content of the acidolysis products were incorrectly labeled from left to right as a, c, b, d. The correct order is d, b, c, a. We have verified the original data, and the corrected figure is presented below.


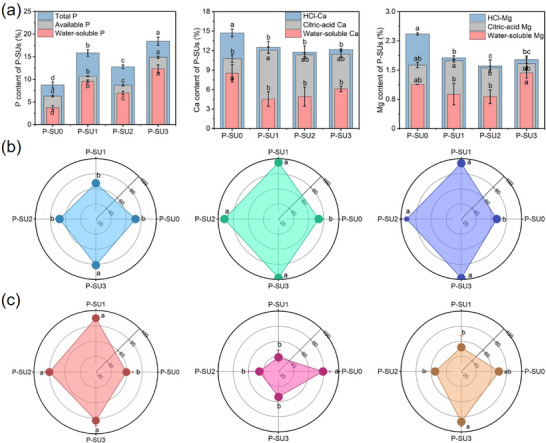


In Figure 10b,c, the units for soil exchangeable Ca and Mg were incorrectly given as mg·kg^−1^ during proofreading. The correct units are mg·g^−1^. We have verified the original data, and the corrected figures are presented below.


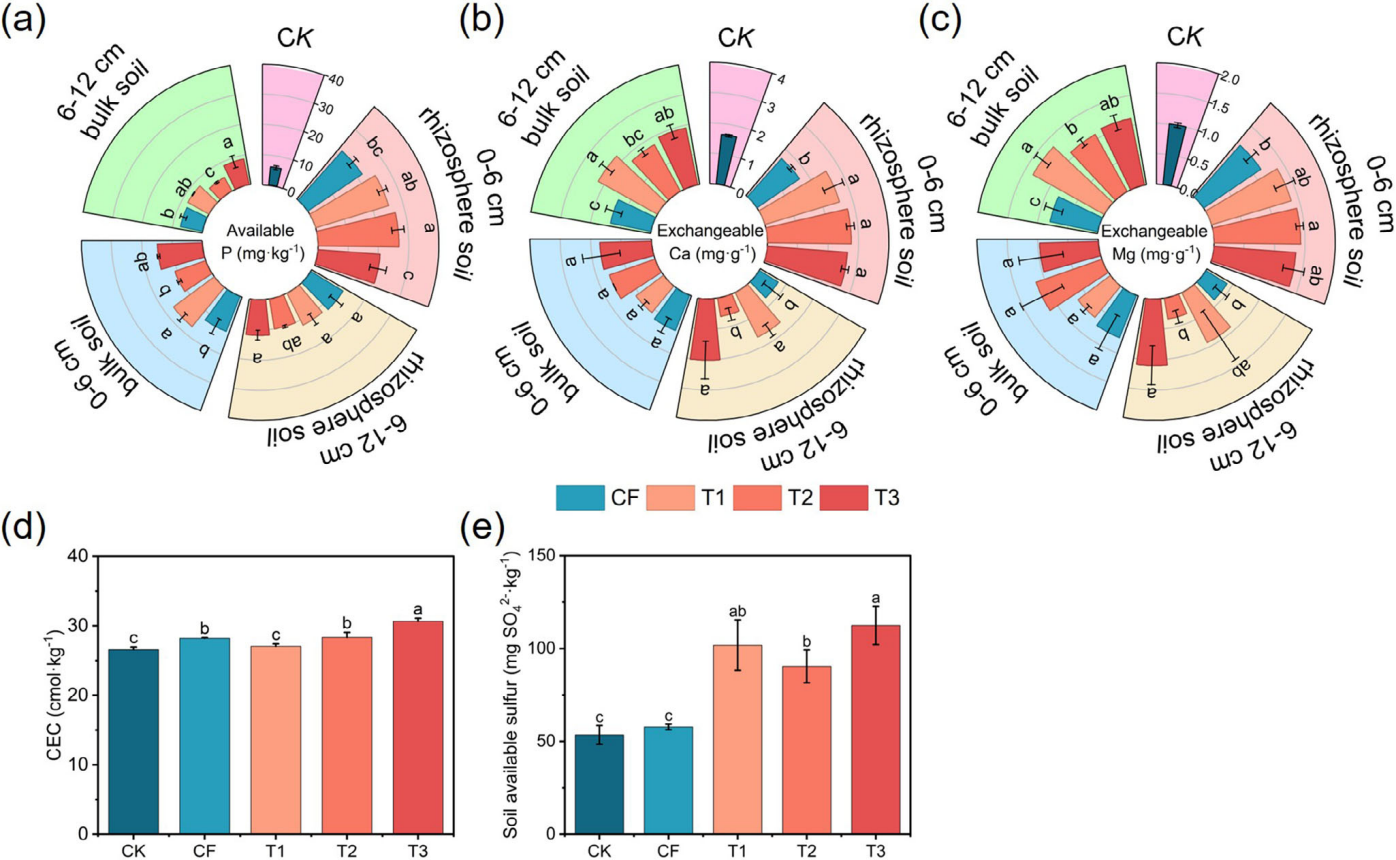


These corrections do not affect the overall findings or conclusions of the study.

