# Supplementary material for: Multifunctional Tailoring of Fertilizer Composites Directly Derived From Phosphate Rock
Source: Adv Sci (Weinh). 2025 Dec 23;13(11):e17533. doi: 10.1002/advs.202517533 (PMC12931167; doi:10.1002/advs.202517533)
Supplement: Supplementary file 1 — Supporting Information [file ADVS-13-e17533-s001.docx]

**Supporting information**

**Multifunctional Tailoring of Fertilizer Composites Directly Derived from Phosphate Rock**

**Author names and affiliations:** Zenglian Qi **^1^**, Jianchao Wang **^1^**, Lulu Chen **^1^**, Zhenya Lu **^1^**, Guodong Wang **^2^**, Hang Ma **^2^**, Cuihong Hou **^3^**, Xinxin Wang **^4^**, Wenqi Ma **^5^**, Changzhou Wei **^6^**, Jianbo Shen **^1^**, Fusuo Zhang **^1^**, Minghao Zhuang ***^, 7^**, Chengdong Huang ***^, 1^**

**^1^** State Key Laboratory of Nutrient Use and Management; Key Laboratory of Plant-Soil Interactions, Ministry of Education; National Academy of Agriculture Green Development; National Observation and Research Station of Agriculture Green Development (Quzhou, Hebei); College of Resources and Environmental Sciences, China Agricultural University, Beijing, 100193, China.

**^2^** Research and Development Center, Yunnan Yuntianhua Co., Ltd., Kunming, 650228, China.

**^3^** School of Chemical Engineering, Zhengzhou University, Zhengzhou, 450001, China

**^4^** College of Horticulture, Hebei Agricultural University, Baoding, 071001, China

**^5^** College of Resources and Environmental Science, Hebei Agricultural University, Baoding, 071000, China

**^6^** Agricultural College, Shihezi University, Shihezi, 832000, China.

**^7^** State Key Laboratory of Urban and Regional Ecology, Research Center for Eco-Environmental Sciences, Chinese Academy of Sciences, Beijing 100085, China

*****Corresponding Author E-mail: mhzhuang@rcees.ac.cn

chengdonghuang@cau.edu.cn

**Supporting information**

**Figures:**

**Figure S1.** Photographs of the P-SUs

**Figure S2.** Sampling method for soil from pot experiments

**Figure S3.** SEM images of acidolysis products from (a) P-SU0, (b) P-SU1, (c) P-SU2, and (d) P-SU3 at low magnification

**Figure S4.** Parameters of the Korsmeyer-Peppas model fitting the nutrient release kinetics of MFCs under different conditions

**Figure S5.** Leaching dynamics of water-soluble nutrients from MFCs

**Figure S6.** Effect of MFCs treatments on root fresh weight of Chinese cabbage

**Figure S7.** Effect of MFCs treatments on leaf SPAD value of Chinese cabbage

**Figure S8.** Soil EC under different MFCs treatments

**Figure S9.** Heavy metal risk assessment of MFCs

**Figure S10.** Heavy metal risk assessment of soil

**Figure S11.** Heavy metal risk assessment of Chinese cabbage leaves

**Figure S12.** Pore structure distribution in MFCs

**Figure S13.** XRD patterns of LPR from different batches

**Figure S14.** Relationship of the pore structure of MFCs with its Korsmeyer-Peppas release constant (k)

**Tables:**

**Table S1.** Heavy metal content in the LPR used in this study

**Table S2.** Particle size distribution of the P-SUs

**Table S3.** Porosity characteristics of the MFCs

**Table S4.** Effects of different MFCs treatments on the growth parameters of Chinese cabbage

**Table S5.** Assessment of resource consumption and solid waste emission for MFCs production


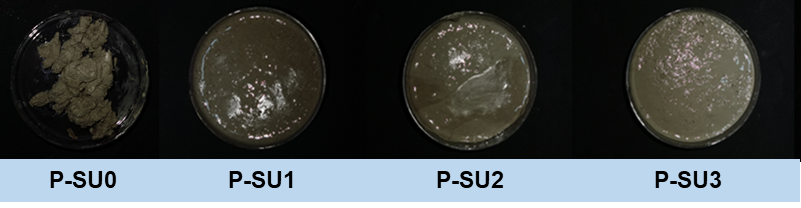


Figure S1. Photographs of the P-SUs


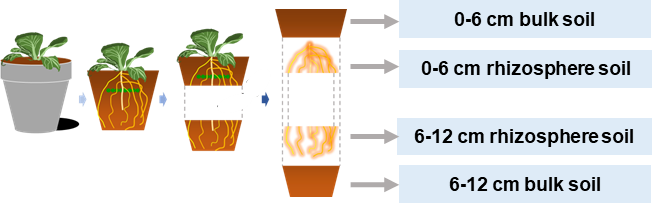


Figure S2. Sampling method for soil from pot experiments


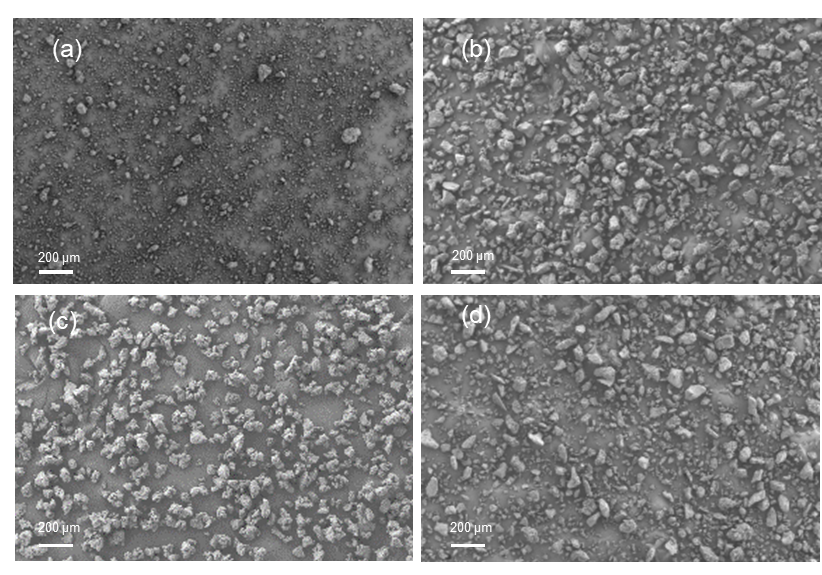


Figure S3. SEM images of acidolysis products from (a) P-SU0, (b) P-SU1, (c) P-SU2, and (d) P-SU3 at low magnification


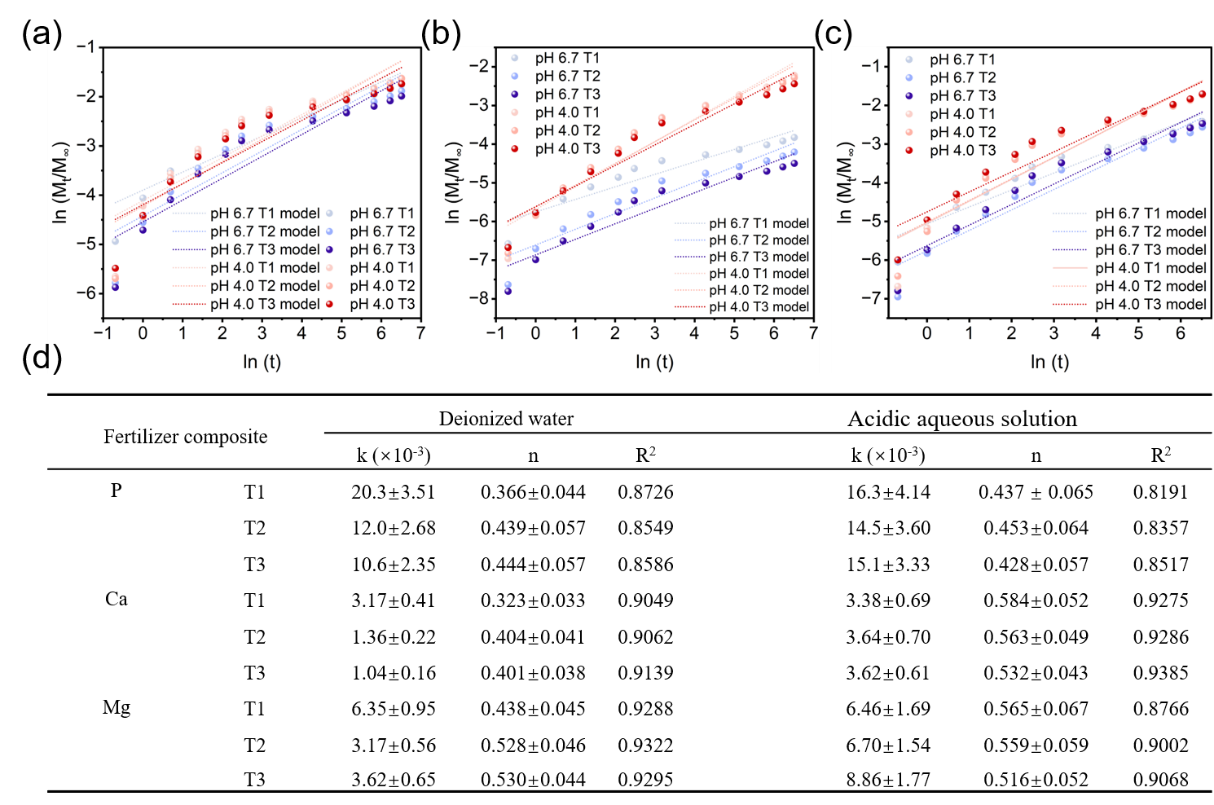


Figure S4. Parameters of the Korsmeyer-Peppas model fitting the nutrient release kinetics of MFCs under different conditions. (a) P; (b) Ca and (c) Mg. Data are presented as mean ± SD (n = 3).


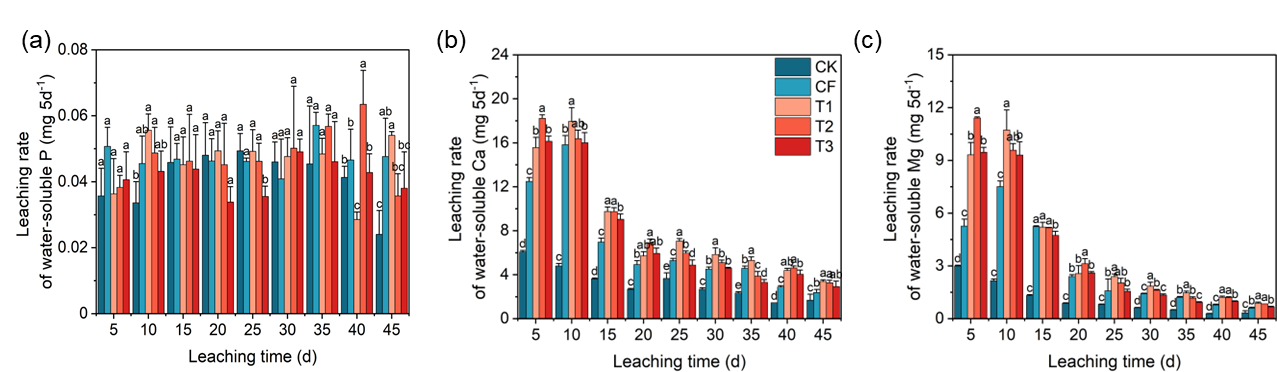


Figure S5. Leaching dynamics of water-soluble nutrients from MFCs: (a) P, (b) Ca, and (c) Mg. Data are presented as mean ± SD (n = 3). Significance was determined by one-way ANOVA followed by Duncan’s test. Different lowercase letters above the bars indicate significant differences among groups at p < 0.05.


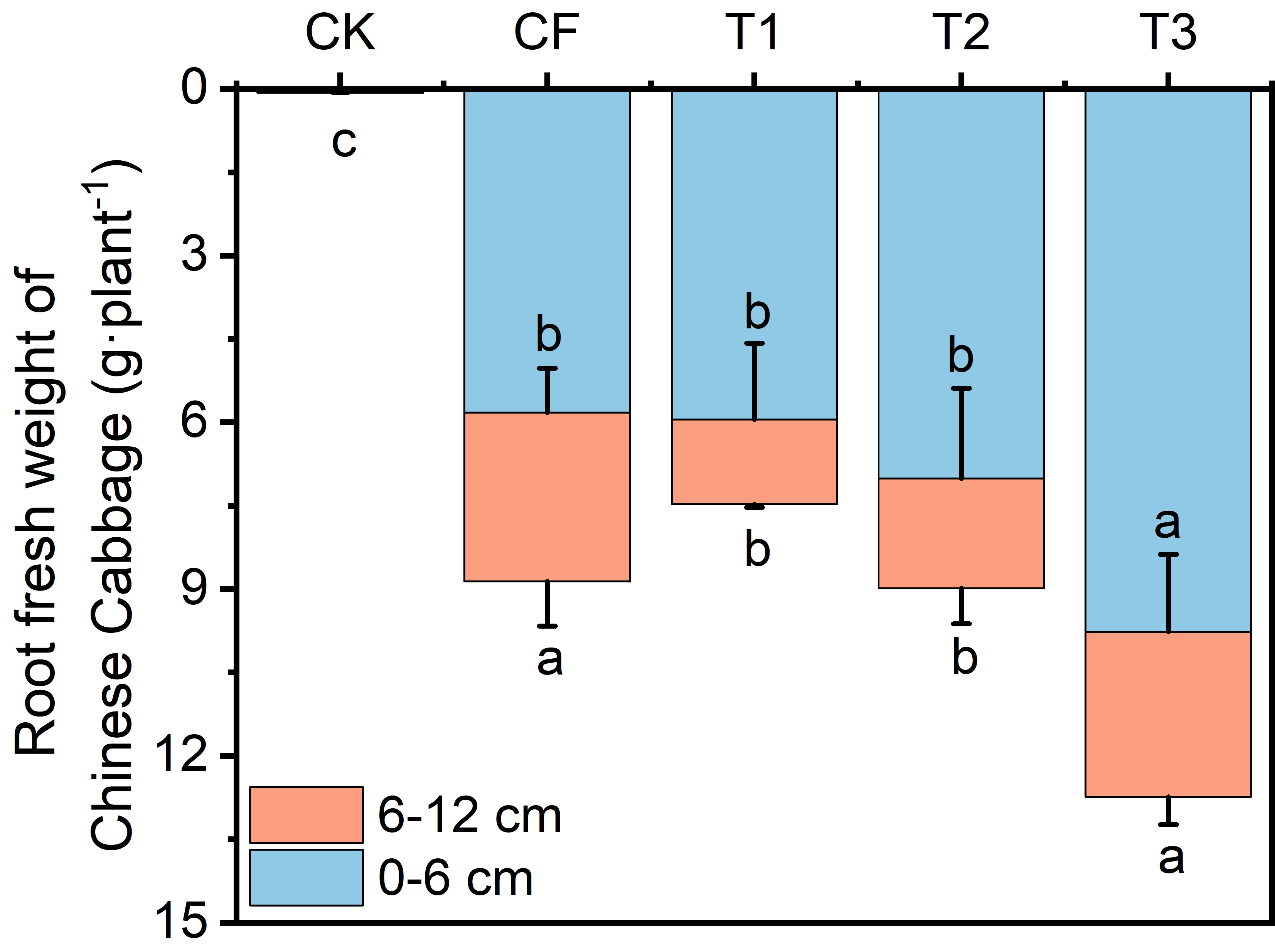


Figure S6. Effect of MFCs treatments on root fresh weight of Chinese cabbage. Data are presented as mean ± SD (n = 3). Significance was determined by one-way ANOVA followed by Duncan’s test. Different lowercase letters above the bars indicate significant differences among groups at p < 0.05.


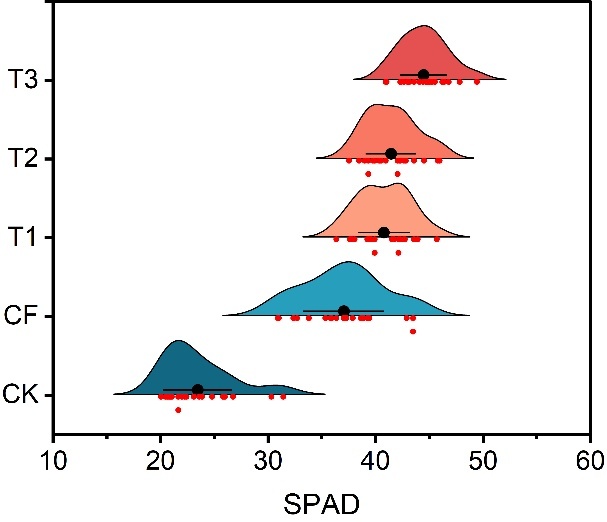


Figure S7. Effect of MFCs treatments on leaf SPAD value of Chinese cabbage


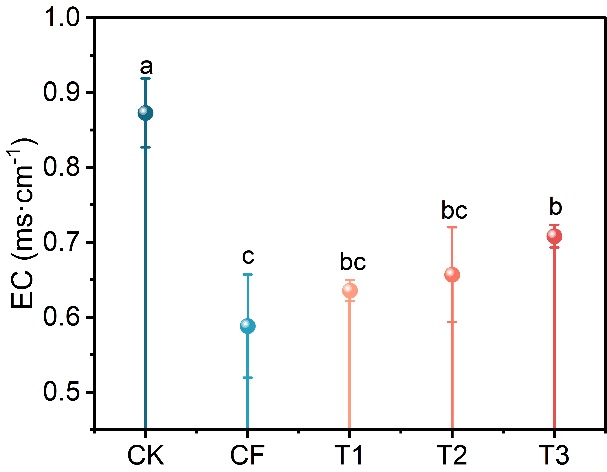


Figure S8. Soil EC under different MFCs treatments. Data are presented as mean ± SD (n = 3). Significance was determined by one-way ANOVA followed by Duncan’s test. Different lowercase letters above the bars indicate significant differences among groups at p < 0.05.


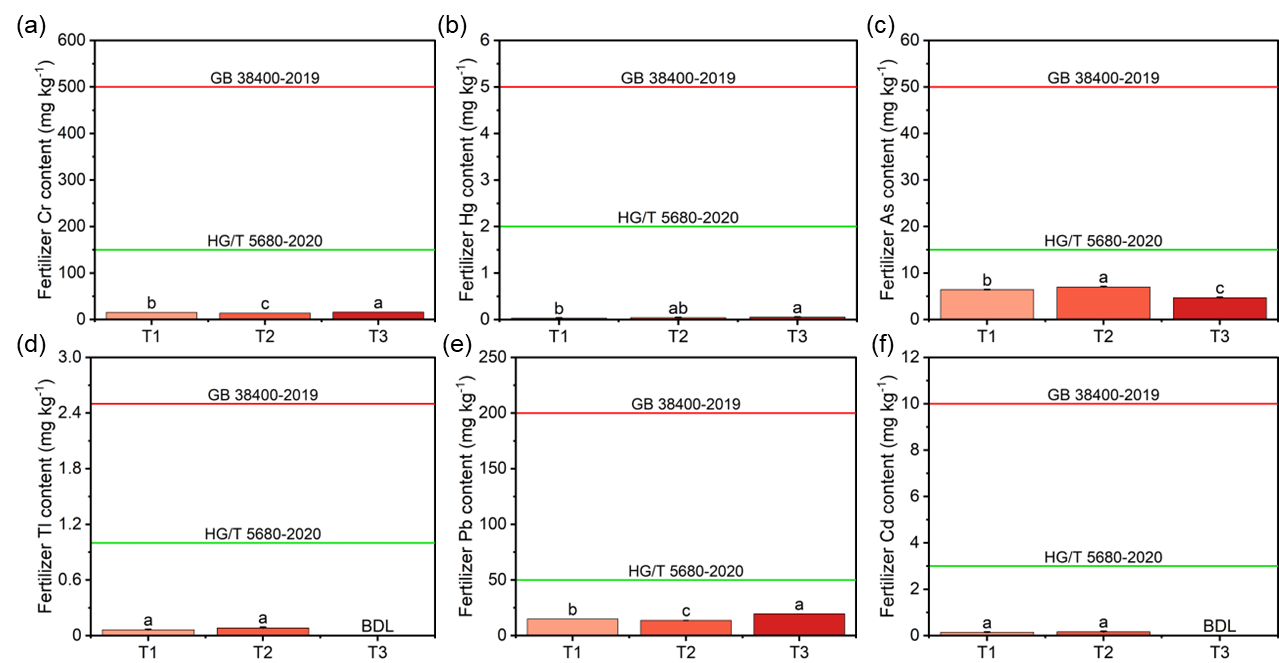


Figure S9. Heavy metal risk assessment of MFCs. Concentrations of (a) Cr, (b) Hg, (c) As, (d) Tl, (e) Pb, and (f) Cd in treatments T1, T2, and T3. The red and green dashed lines indicate the maximum permissible limits according to Chinese national standards (GB 38400-2019 and HG/T 5680-2020). BDL: below detection limit. Data are presented as mean ± SD (n = 3). Significance was determined by one-way ANOVA followed by Duncan’s test. Different lowercase letters above the bars indicate significant differences among groups at p < 0.05.


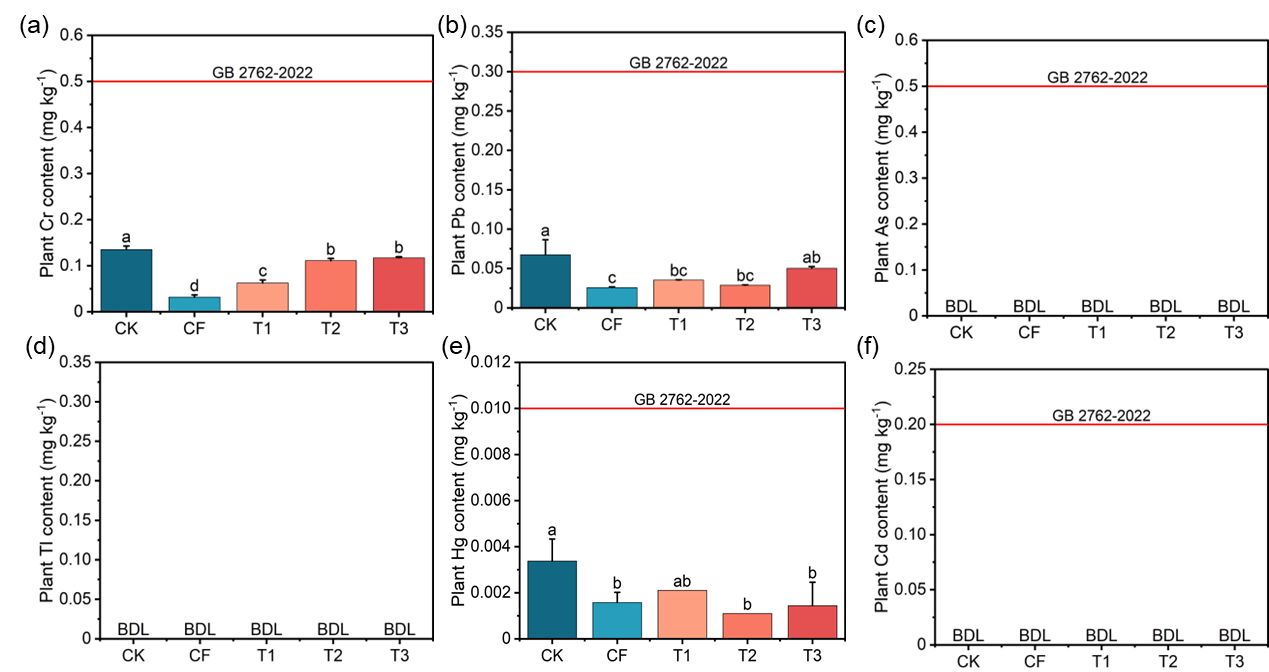


Figure S10. Heavy metal risk assessment of soil. Concentrations of (a) Cr, (b) Pb, (c) As, (d) Tl, (e) Hg, and (f) Cd in soil under different treatments. The red dashed lines indicate the safety limits set by the Chinese standard (GB/T 15618-2018). BDL: below detection limit. Data are presented as mean ± SD (n = 3). Significance was determined by one-way ANOVA followed by Duncan’s test. Different lowercase letters above the bars indicate significant differences among groups at p < 0.05.


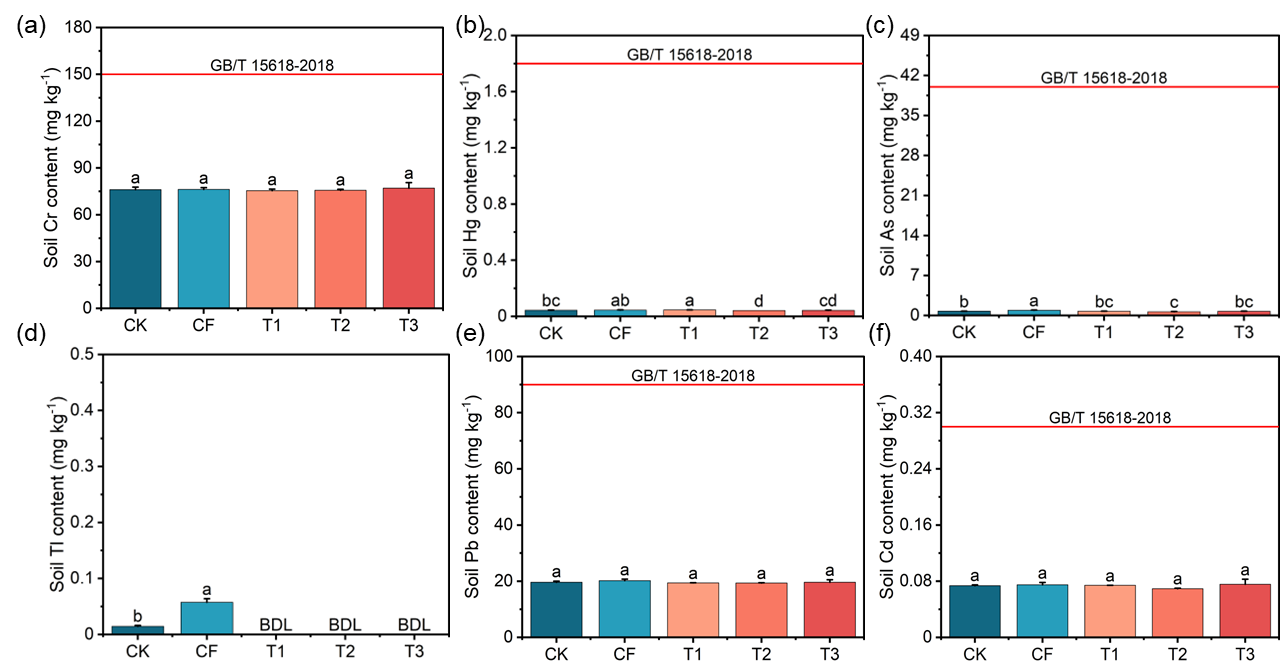


Figure S11. Heavy metal risk assessment of Chinese cabbage leaves. Concentrations of (a) Cr, (b) Hg, (c) As, (d) Tl, (e) Pb, and (f) Cd in leaves. The red dashed lines indicate the safety limits established by the Chinese national standard (GB 2762-2022). BDL: below detection limit. Data are presented as mean ± SD (n = 3). Significance was determined by one-way ANOVA followed by Duncan’s test. Different lowercase letters above the bars indicate significant differences among groups at p < 0.05.


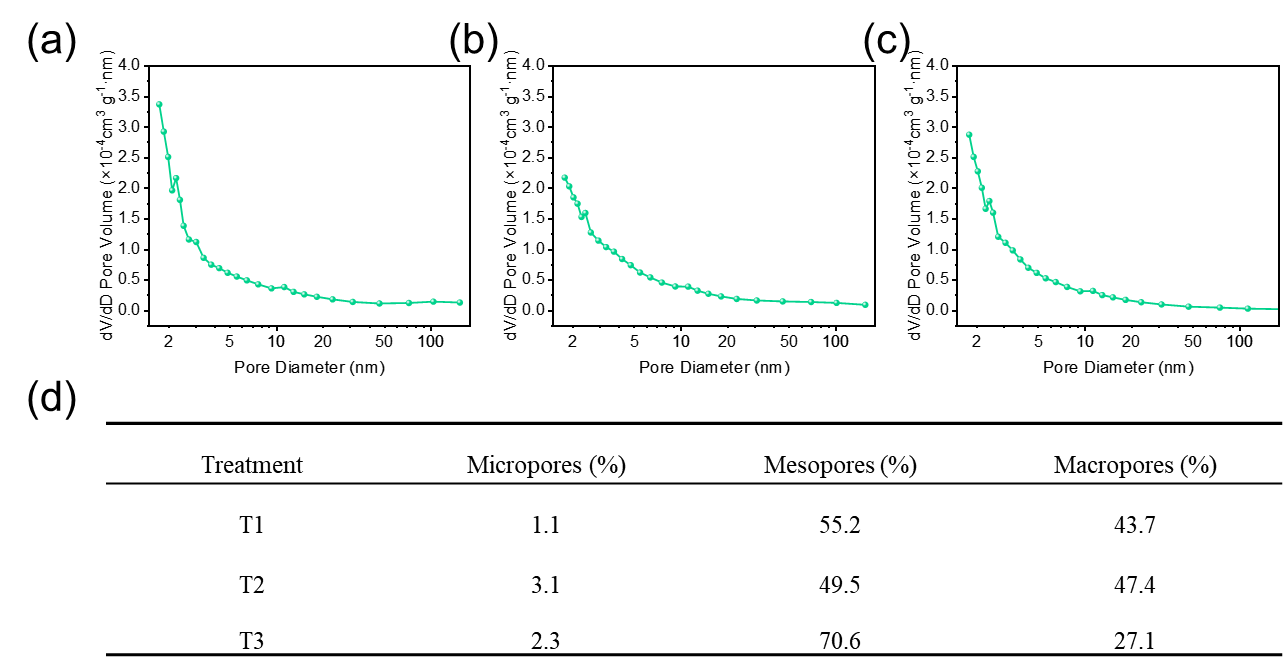


Figure S12. Pore structure distribution in MFCs


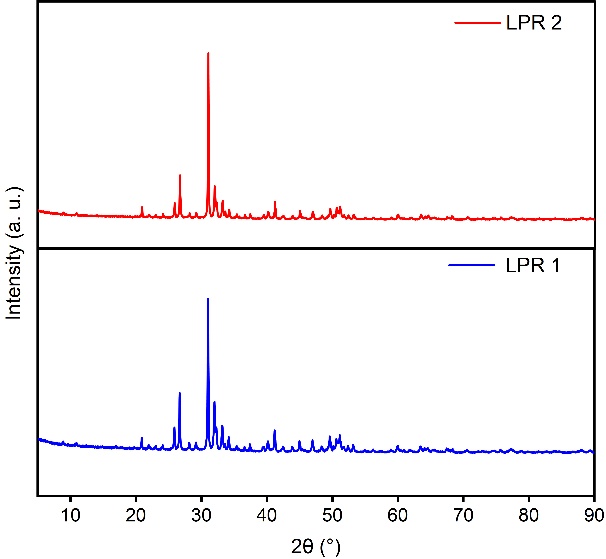


Figure S13. XRD patterns of LPR from different batches. LPR1 is the sample used in this study, LPR2 is the identifier for a second batch of phosphate rock.


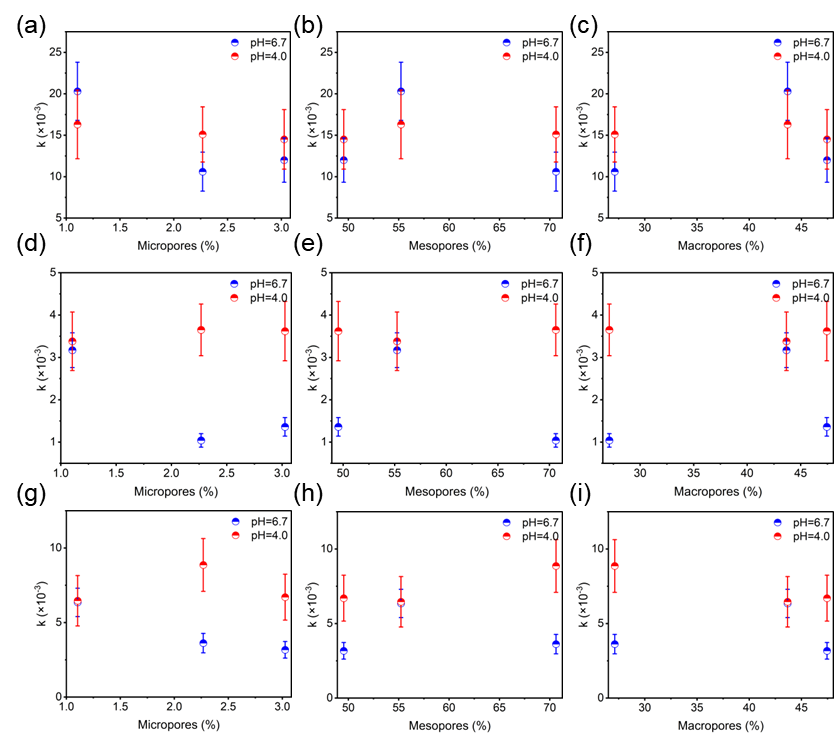


Figure S14. Conducted a detailed analysis to explore the relationship between pore structure and release kinetics. (a-c) Micropores - k (P, Ca, Mg) at different pH; (d-f) Mesopores - k (P, Ca, Mg) at different pH; (g-i) Macropores - k (P, Ca, Mg) at different pH.

Table S1. Heavy metal content (mg·kg^-1^) in the LPR used in this study

| Heavy metal | Pb | Cr | Tl | Hg | As |
| --- | --- | --- | --- | --- | --- |
| LPR | 52.0 | 14.7 | 0.1 | 0.2 | 20.0 |

| Treatment | D (10)  (μm) | D (50)  (μm) | D (90)  (μm) |
| --- | --- | --- | --- |
| P-SU0 | 2.16 | 7.54 | 24.17 |
| P-SU1 | 2.55 | 30.97 | 95.65 |
| P-SU2 | 8.42 | 42.35 | 105.13 |
| P-SU3 | 7.87 | 41.95 | 92.88 |

Table S2. Particle size distribution of the P-SUs

Table S3. Porosity characteristics of the MFCs

| Thermophysical properties | T1 | T2 | T3 |
| --- | --- | --- | --- |
| BET surface area (m^2^·g^-1^) | 0.8800 | 0.9687 | 1.1979 |
| t-plot micropore area (m^2^·g^-1^) | 0.2020 | 0.1015 | 0.4414 |
| Pore volume (cm^3^·g^-1^) | 0.001886 | 0.003315 | 0.003478 |

| Treatment | Number of leaves  (# per plant) | Leaf length  (cm) | Leaf width  (cm) |
| --- | --- | --- | --- |
| CK | 4.0±0 d | 5.0±0.2b | 2.7±0.1 c |
| CF | 9.7±1.5 c | 25.8±1.4 a | 14.7±1.9 b |
| T1 | 12.0±1.0 b | 25.6±0.6 a | 15.3±1.1 b |
| T2 | 12.7±0.6 b | 26.9±0.8 a | 16.2±1.1 ab |
| T3 | 15.3±0.6 a | 27. 7±1.9 a | 17.7±0.3 a |

Table S4. Effects of different MFCs treatments on the growth parameters of Chinese cabbage

Note: Data are presented as mean ± SD (n = 3). Different lowercase superscript letters within the same column indicate significant differences among treatments according to Duncan’s new multiple range test at p < 0.05.

Table S5. Assessment of resource consumption and solid waste emission for MFCs production

| Treatment | LPR (t) | Phosphate tailing (t) | Phosphogypsum (t) |
| --- | --- | --- | --- |
| CK | 0 | 0 | 0 |
| CF | 0.67 | 0.31 | 0.56 |
| T1 | 0.65 | 0.27 | 0.49 |
| T2 | 0.66 | 0.28 | 0.51 |
| T3 | 0.65 | 0.25 | 0.45 |

Note: Material flow for producing 1 t of 17-10-11 MFCs, showing consumption of LPR and generation of phosphate tailing and phosphogypsum.
